# Extended Analysis and Evidence Integration of Chloroprene as a Human Carcinogen

**DOI:** 10.1111/risa.13397

**Published:** 2019-09-16

**Authors:** Sonja N Sax, P. Robinan Gentry, Cynthia Van Landingham, Harvey J. Clewell, Kenneth A. Mundt

**Affiliations:** ^1^ Ramboll US Corporation Amherst MA USA; ^2^ Ramboll US Corporation Monroe LA USA; ^3^ Ramboll US Corporation Research Triangle Park NC USA; ^4^ Cardno Chemrisk Boston MA USA

**Keywords:** Cancer inhalation unit risk, chloroprene, evidence integration, interspecies extrapolation, pharmacokinetic modeling

## Abstract

β‐Chloroprene is used in the production of polychloroprene, a synthetic rubber. In 2010, Environmental Protection Agency (EPA) published the Integrated Risk Information System “Toxicological Review of Chloroprene,” concluding that chloroprene was “likely to be carcinogenic to humans.” This was based on findings from a 1998 National Toxicology Program (NTP) study showing multiple tumors within and across animal species; results from occupational epidemiological studies; a proposed mutagenic mode of action; and structural similarities with 1,3‐butadiene and vinyl chloride. Using mouse data from the NTP study and assuming a mutagenic mode of action, EPA calculated an inhalation unit risk (IUR) for chloroprene of 5 × 10^−4^ per µg/m^3^. This is among the highest IURs for chemicals classified by IARC or EPA as known or probable human carcinogens and orders of magnitude higher than the IURs for carcinogens such as vinyl chloride, benzene, and 1,3‐butadiene. Due to differences in pharmacokinetics, mice appear to be uniquely responsive to chloroprene exposure compared to other animals, including humans, which is consistent with the lack of evidence of carcinogenicity in robust occupational epidemiological studies. We evaluated and integrated all lines of evidence for chloroprene carcinogenicity to assess whether the 2010 EPA IUR could be scientifically substantiated. Due to clear interspecies differences in carcinogenic response to chloroprene, we applied a physiologically based pharmacokinetic model for chloroprene to calculate a species‐specific internal dose (amount metabolized/gram of lung tissue) and derived an IUR that is over 100‐fold lower than the 2010 EPA IUR. Therefore, we recommend that EPA's IUR be updated.

## 1 INTRODUCTION

β‐Chloroprene (2‐chloro‐1,3‐butadiene, CAS# 126‐99‐8) is used in the production of polychloroprene (Neoprene). In 2010, the US Environmental Protection Agency (EPA) published the “Toxicological Review of Chloroprene” (Environmental Protection Agency [EPA], 2010), hereafter the 2010 Review, in which EPA derived an inhalation unit risk (IUR) for chloroprene. In the 2010 Review, EPA concluded that chloroprene was “likely to be carcinogenic to humans” based on (1) statistically significant and dose‐related information from a National Toxicology Program (NTP) (National Toxicology Program [NTP], 1998) chronic inhalation bioassay demonstrating the early appearance of tumors, development of malignant tumors, and the occurrence of multiple tumors within and across animal species; (2) evidence of an association between liver cancer risk and occupational exposure; (3) suggestive evidence of an association between lung cancer risk and occupational exposure; (4) a proposed mutagenic mode of action (MOA); and (5) structural similarities between chloroprene and known human carcinogens (1,3‐butadiene and vinyl chloride) (EPA, 2010).

In the 2010 Review, the EPA derived an IUR for lifetime exposure to chloroprene of 5 × 10^−4^ per microgram per cubic meter (µg/m^3^). This is among the top 10 highest IURs generated by EPA for any chemical classified by EPA or the International Agency for Research on Cancer (IARC) as a known or likely/probable human carcinogen (not including carcinogenic metals or coke oven emissions). The IUR also is orders of magnitude greater than those for the known human carcinogens—vinyl chloride, 1,3‐butadiene, and benzene. As a result of the high IUR, EPA's (2011) National Air Toxics Assessment (NATA) identified LaPlace, Louisiana, as having the highest excess cancer risk in the nation, estimated at eight per 10,000, specifically due to chloroprene emissions in the area (EPA, 2011).

To understand how EPA arrived at such a high IUR, we conducted an updated weight of evidence (WOE) analysis for chloroprene including a critical review of the epidemiological and toxicological evidence for chloroprene carcinogenicity. This new analysis incorporates the evidence integration approach recommended by the National Research Council (NRC) of the National Academies of Science and published in a series of reports that reviewed the Integrated Risk Information System (IRIS) process (National Research Council [NRC], 2011, 2014). It also serves as a case study that responds to the current interest in developing approaches for the integration of evidence from diverse information streams. In addition, we calculated a chloroprene IUR using standard EPA methodology, published pharmacokinetic data, and output from published physiologically based pharmacokinetic (PBPK) models, and we compared our findings to those of the EPA.

## 2 LITERATURE SEARCH AND REVIEW

We conducted a comprehensive search to identify literature specific to the potential carcinogenicity of chloroprene to ensure that the most recent literature was considered. We conducted the literature search using the National Institutes of Health United States, National Library of Medicine PubMed, and Toxline databases. Searches on PubMed were performed in DistillerSR, a reference management and data abstraction tool, and ToxLine searches were imported into DistillerSR following completion.

**Figure 1:**
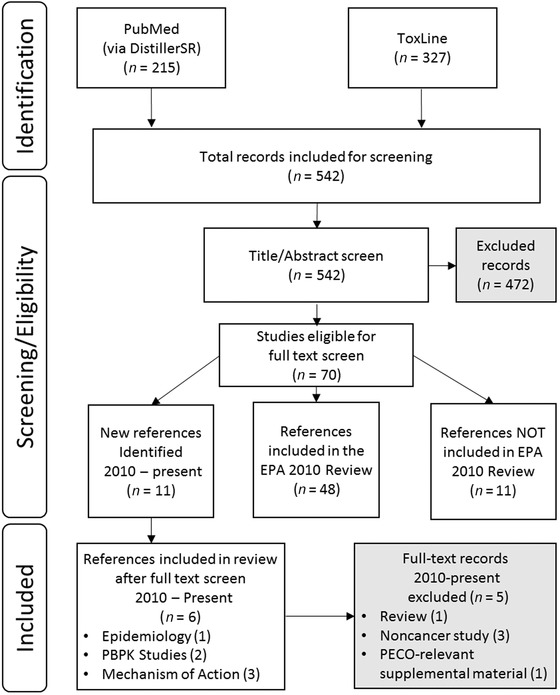
Results from literature search and screening.

The keywords used for the literature searching are provided in Table I. The PubMed search query yielded 215 articles and the Toxline database search yielded 327 articles. The titles and abstracts of each study were screened separately by two individuals for eligibility of inclusion under the defined Populations of interest, Exposures, Comparators, and Outcomes (PECO) criteria for this evaluation (Table II). The inclusion criteria were the identification of studies that evaluated the potential carcinogenicity of chloroprene. This included studies conducted in humans, animals, or cells, as well as studies conducted to develop PBPK models. We excluded studies focused on the evaluation of other health outcomes and the potential association with chloroprene exposures. Fig. 1 provides the details of the literature search and screening results. Of the initial 542 studies identified, 70 studies fit the inclusion criteria after screening of titles and abstracts. Of these, 48 studies had been addressed in the EPA (2010) Review as part of the evaluation of the potential for carcinogenicity; an additional 11 studies were published prior to the 2010 Review and were not included in the 2010 Review, and 11 new studies (published after the 2010 Review) were identified. This assessment includes a critical review of all 48 studies that were considered in the 2010 Review, as well as all of the 22 additional studies that were available prior to the 2010 Review or published subsequent to the 2010 Review.

**Table I:** Literature Search Keyword Search Strings

PubMed	((“Chloroprene” OR “1,3‐Butadiene, 2‐chloro‐” OR “2‐Chloor‐1,3‐butadieen” OR “2‐Chlor‐1,3‐butadien” OR “2‐Chlorbuta‐1,3‐dien” OR “2‐chloro‐1,3‐butadiene” OR “2‐Chloro‐1,3‐butadiène” OR “2‐chlorobuta‐1,3‐diene” OR “Chloropren”))	Results: 215
TOXLINE	(chloroprene OR “2 chlorobutadiene” OR “cloroprene italian ” “chloropreen dutch” chlorobutadiene OR 126‐99‐8 [rn]) [not] PubMed [org] [not] pubdart [org]	Results: 327

**Table II:** Population, Exposure, Comparator, and Outcome (PECO) Framework used for Assessment

PECO Element	Evidence
Population	**Human**: Any population (occupational, general population, including children and other sensitive population). The following study designs will be considered most informative: controlled exposure, cohort, case‐control, or cross‐sectional. Note: Case reports and case series will be tracked during study screening but are not the primary focus of this assessment.
	**Animal**: Nonhuman mammalian animal species (whole organism) of any life stage.
	**Model systems/*in vitro*/*in silico***: Human or animal cells, tissues, or biochemical reactions (e.g., ligand binding assays) with *in vitro* exposure regimens; bioinformatics pathways of disease analysis; or high‐throughput screening data. These studies are tagged during title and abstract/full‐text screening and an iterative approach is used to prioritize for further analysis based on likelihood of the study to impact hazard conclusions or inform toxicity value derivation. Studies that do not undergo further analysis will be classified as PECO‐relevant supplemental information.
Exposure	Exposure based on administered dose, or concentration, or biomonitoring data.The potential for human exposure to chloroprene primarily is via inhalation and perhaps by the dermal route. ADME and PBPK studies will also be included. Relevant forms are listed below: Chloroprene (CASRN 126‐99‐8) or its metabolites such as (1‐chloroethenyl)oxirane or (2‐chloro‐2‐ethenyl)oxirane. Mixture studies will be included if they include a chloroprene‐only group (or one of its metabolites).
Comparator	**Human**: A comparison, or reference population exposed to lower levels (or no exposure/exposure below detection levels), or to chloroprene for shorter periods of time.
	**Animal and *in vitro***: Quantitative exposure versus lower or no exposure with concurrent vehicle control group.
Outcome	Only carcinogenic health outcomes; ADME and PBPK studies.

Of the 11 pre‐2010 studies identified (Ambartsumyan, Khapkina, & Agoyan, 1968; Avakian, Gasparian, Avetisian, & Kondakova, 1956; Clary, 1977; Lewis, Ioannides, & Parke, 1996; Lloyd, 1976; Melnick & Khon, 2000; Morgan, 1997; Rannug & Ostman, 1983; Valentine & Himmelstein, 2001; Zaridze, Bulbulyan, Changuina, Margaryan, & Boffetta, 2001; Zhang, Sussman, Macina, Rosenkranz, & Klopman, 1996), we excluded two studies (Ambartsumyan et al., 1968; Avakian et al., 1956) because they were in Russian and the screening of the available titles indicated that they did not fit the inclusion criteria. Only the abstract for Morgan (1997) was available and it indicated that the study was a summary of the results of a six‐month NTP inhalation study conducted in two strains of transgenic mice; however, no technical report for the study was available from the NTP website. The available information is discussed in Section 3.1. Three of the studies identified (Clary, 1977; Lloyd, 1976; Valentine & Himmelstein, 2001) were excluded because they were presentations, reviews, or commentaries and did not provide any new information not already considered in the 2010 Review or for this assessment. The studies by Lewis et al. (1996) and Zhang et al. (1996) both attempted to predict the potential carcinogenicity for multiple compounds, including chloroprene, using the available data from NTP and predictive models, based on molecular structure and comparative analyses. As these studies did not rely on new information and were focused on the NTP results for chloroprene, we also excluded these studies from further consideration. Melnick and Kohn (2000) describe dose–response analyses of 1,3‐butadiene, chloroprene, and isoprene using NTP data; however, the authors describe PBPK models for 1,3‐butadiene and isoprene only. As this study relied primarily on NTP data, and did not provide any PBPK modeling of chloroprene, we excluded it. The study by Rannug and Ostman (1983) was also excluded because it tested the mutagenicity of vulcanized rubber fumes that had been cured at high temperatures, collected on filters and extracted with acetone, and did not test chloroprene. Finally, the study by Zaridze et al. (2001) was excluded as a review of the study indicated that it is a reanalysis of the Armenian and Russian cohorts described by Bulbulyan et al. (1998) and Bulbulyan et al. (1999) and the same limitations apply to this study as those discussed in Section 4.

Of the 11 studies published after the 2010 Review, we found that four studies did not meet our inclusion criteria (Chen et al., 2017; Eckert, Leng, Gries, & Goen, 2012; NTP, 2016; Rickert, Hartung, Kardel, Teloh, & Daldrup, 2012) and were, therefore, excluded from this assessment. One was the summary profile from the 14th Report on Carcinogens (NTP, 2016) and was excluded because it did not provide any information that was not already considered in the 2010 Review or this assessment. The study by Rickert et al. (2012) was excluded because it is an acute exposure, poisoning study and the Chen et al. (2017) study was excluded because it has no assays or experiments focusing on evaluating the potential for cancer or endpoints relevant to cancer following chloroprene exposure. The paper by Eckert et al. (2012) was an analytical method article for measuring biomarkers of exposure to chloroprene in urine and was, therefore, excluded.

Seven studies identified post‐2010 met our inclusion. These included one epidemiological study (Garcia, Hurley, Nelson, Hertz, & Reynolds, 2015), which we determined to be irrelevant because of the ecological nature of the study and lack of information on exposure of the study population specifically to chloroprene. The other six studies included three PBPK or absorption, distribution, metabolism and elimination (ADME) studies (Allen et al., 2014; Eckert, Leng, Gries, & Goen, 2013; Yang, Himmelstein, & Clewell, 2012), and three mechanistic studies (Guo & Xing, 2016; Thomas et al., 2013; Wadugu, Ng, Bartley, Rowe, & Millard, 2010). We excluded the study by Eckert et al. (2013) from further consideration as this was a pilot study focused on characterizing exposure to chloroprene through biomonitoring of workers to identify metabolites of exposure to chloroprene. Reviews of the studies that met the inclusion criteria are provided in the following sections.

## 3 TOXICOLOGICAL EVIDENCE

In this section, we describe the toxicological evidence for chloroprene carcinogenicity first discussing the evidence in animal studies, followed by discussion of the *in vitro* or mechanistic evidence. In addition, we review the evidence for the toxicity MOA of chloroprene, followed by information on the toxicokinetics across species.

### 3.1 Animal Studies

Two animal studies evaluated the chronic toxicity and carcinogenicity of chloroprene: a two‐year inhalation study conducted by the NTP (1998) and an 18‐month inhalation study by Trochimowicz, Loser, Feron, Clary, and Valentine (1998). The study protocols are summarized in Table III and results are summarized in Tables IV and V. The NTP (1998) study evaluated effects of chloroprene exposures in B6C3F1 mice and F344/N rats at exposures of 0 ppm, 12.8 ppm, 32 ppm, and 80 ppm (Table III) and reported statistically significant increases in tumor incidence at multiple sites in the B6C3F1 mouse, including all organs (hemangiomas and hemangiosarcomas), lung (bronchiolar/alveolar adenomas and carcinomas), forestomach, Harderian gland (adenomas and carcinomas), kidney (adenomas), skin, liver, and mammary glands (Table IV). With increasing exposures, the tumors generally appeared earlier, and statistically significant pairwise comparisons were reported with increasing exposure level (12.8 ppm, 32 ppm, or 80 ppm ppm). In the NTP (1998) study, the incidence of lung tumors was statistically significantly elevated at the lowest exposure tested (12.8 ppm) in both female and male mice compared to unexposed controls (0 ppm). Statistically significantly increased lung tumor incidence was not observed in male and female rats administered chloroprene at concentrations up to 80 ppm compared to unexposed controls (0 ppm). F344/N rats had significantly fewer tumors observed in fewer locations than the B6C3F1 mice. These tumors typically were observed only at the highest chloroprene exposure level (>32 ppm) (Table IV).

**Table III:** Study Protocols for *in vivo* Animal Toxicity Studies

Reference	Species	Number of Animals in Each Group	Dosing Groups (ppm)	Duration
Morgan (1997)	Tg.AC (FVB/N); Tg.Lac1/C57BL/6 (Big Blue)	20/strain/dose	0, 2, 12.8, 80	6 h/day, 5‐day/wk for 26 weeks
NTP (1998)	F344/N rats	10 M, 10 F	0, 32, 80, 200, or 500	6 h/day, 5‐day/wk for 16 days
	F344/N rats	10 M, 10 F	0, 5, 12, 32, 80, or 200	6 h/day, 5‐day/wk for 13 weeks
	F344/N rats	50 M, 50 F	0, 12.8, 32, or 80	6 h/day, 5‐day/wk for two years
	B6C3F1 mice	10 M, 10 F	0, 12, 32, 80, or 200	6 h/day, 5‐day/wk for 16 days
	B6C3F1 mice	10 M, 10 F	0, 5, 12, 32, or 80	6 h/day, 5‐day/wk for 13 weeks
	B6C3F1 mice	50 M, 50 F	0, 12.8, 32, or 80	6 h/day, 5‐day/wk for two years
Trochimowicz et al. (1998)	Wistar rats	100 M, 100 F	0, 10, or 50	6 h/day, 5‐day/wk for two years
	Syrian hamsters	100 M, 100 F	0, 10, or 50	6 h/day, 5‐day/wk for 18 months

**Table IV:** Key Results from the National Toxicological Program Chronic Chloroprene Inhalation Bioassay, Key Sites, and All Tumors (NTP, 1998)

	B6C3F1 Mice								F344/N Rats							
Site^a^	Males				Females				Males				Females			
**ppm**	**0**	**12.8**	**32**	**80**	**0**	**12.8**	**32**	**80**	**0**	**12.8**	**32**	**80**	**0**	**12.8**	**32**	**80**
**Number**	**50**	**50**	**50**	**50**	**50**	**50**	**50**	**50**	**50**	**50**	**50**	**50**	**50**	**50**	**50**	**50**
Oral cavity									0	2	**5**	**12**	1	3	5	**11**
Thyroid					1	3	1		0	2	**4**	**5**	1	1	1	5
Lung	13	**28**	**36**	**43**	4	**28**	**34**	**42**	2	2	4	6	1	0	0	3
All organs hemangiomas or hemangiosarcomas	3	**14**	**23**	**21**	4	6	**18**	8								
Harderian gland	2	5	**10**	**12**	2	5	3	**9**								
Kidney	0	2	**3**	**9**					1	**8**	**6**	**8**	0	0	0	4
Mammary gland					3	5	8	**12**					28	34	36	36
Forestomach	1		2	5	1			**4**								
Liver	43	38	43	42	20	26	**20**	**30**								
Skin						**11**	**11**	**18**	3	5	3	5				
Mesentery						4	**8**	3								
Zymbal's gland								3								
Hematopoietic																

Bold indicates statistically significant increases compared to controls (0 ppm) reported by NTP (1998) based on logistic regression test (*p* < 0.05).

^a^All observed adenomas and carcinomas (single and step sections) included.

**Table V:** Key Results from Trochimowicz et al. (1998) Chronic Inhalation Study of Chloroprene Exposure

	Wistar Rats						Syrian Hamsters					
**Site**	Males			Females			Males			Females		
**ppm**	**0**	**10**	**50**	**0**	**10**	**50**	**0**	**10**	**50**	**0**	**10**	**50**
**Total number**	**97**	**13**	**100**	**99**	**24**	**100**	**100**	**97**	**97**	**94**	**93**	**97**
Thyroid^a^	15	1	14	14	1	23	5	2	1	2	4	4
Lung				1								
All organs hemangiomas or hemangiosarcomas			1									
Kidney			2	1		1	2					
Mammary gland^b^				34	9	**46**						
Liver				1				2				1
Skin			2					1				
Zymbal's gland						1			1			
Hematopoietic	1		3			1						

Bold indicates statistically significant increases compared to controls (0 ppm) reported by Trochimowicz et al. (1998) based the chi‐squared test (*p* < 0.05).

^a^Thyroid tumors include adenomas and carcinomas.

^b^Mammary tumors include adenomas, fibroadenomas, adenocarcinomas, papillary carcinomas, and unidentified tumors (some animals may have more than one mammary tumor).

An abstract of a six‐month inhalation study conducted by the NTP in two strains of transgenic mice (Tg.AC (FVB/N); Tg.Lac1/C57BL/6 (Big Blue) (Morgan, 1997) was also identified through literature searching. No technical report or information providing results was available on the NTP website (https://ntp.niehs.nih.gov/testing/status/agents/ts‐10573‐d.html). The abstract indicated that the study provided no evidence of carcinogenicity associated with exposure to chloroprene in either strain of transgenic mice.

Trochimowicz et al. (1998) evaluated effects of exposure to chloroprene in Wistar rats and Syrian hamsters at 0 ppm, 10 ppm, and 50 ppm (Table III), and reported large variability in the tumor incidence and sites across species (Table V). Although tumors appeared across multiple sites in both rats and hamsters, there were no statistically significant increases at any particular site (except mammary gland in rats), no significant trends observed with increasing concentration (up to 50 ppm), and tumor incidence of less than 20% in hamsters (Table V).

Across all tested species, mice appear to develop tumors at lower concentrations in response to long‐term chloroprene exposure (75% or greater of lifetime), and lung tumors in mice appear to be the most sensitive endpoint. In the 2010 Review, EPA also concluded that female mice and lung tumors were the most sensitive species and endpoint, respectively. Given the differences in response to chloroprene exposure in mice compared to other laboratory species, it is particularly important to determine whether the mouse is an appropriate model for determining human health risk. If the mouse model is not directly comparable to humans, then using appropriate methods to correct for differences in cross‐species response to chloroprene exposure is critical to the derivation of a valid IUR.

### 3.2 Mechanistic Studies

Chloroprene mutagenicity has been evaluated in both *in vitro* studies (using different exposure systems) in bacteria and in mammalian cells, and *in vivo* studies (NTP, 1998; Shelby, 1990; Shelby & Witt, 1995; Tice, 1988; Tice et al., 1988). The majority of the conventional genetic toxicology studies did not report positive results following administration of chloroprene. For example, the NTP (1998) study states, “chloroprene was not mutagenic in any of the tests performed by the NTP.” In addition, the results of the bacterial mutagenicity studies are equivocal (e.g., NTP, 1998; Pagan, 2007). As shown in Table VI, the results from two studies failed to show any increase in TA1535 or TA100 revertants, whereas chloroprene appeared to be mutagenic in *Salmonella typhimurium* TA100 and/or TA1535, with the addition of S9 mix, which incorporates chloroprene metabolism (Bartsch, Malaveille, Barbin, & Planche, 1979; Willems, 1980). Chloroprene was not mutagenic in *S. typhimurium* strains TA98 or TA1537 (Zeiger et al., 1987).

**Table VI:** Ames Test Results for Chloroprene with TA1535 and/or TA100

			Response	
Study	Method	Exposure	With S9 mix	Without S9 mix
Bartsch et al. (1979)	Desiccator^a^	Four hours	++	+
Westphal et al. (1994)	Pre‐inc^b^	Two hours	−	−
NTP (1998)	Pre‐inc^b^	20 minutes	−	−
Willems (1980)	Desiccator^a^	24–48 hours	++	+

a Plates sealed in desiccator at 37 °C with tops removed.

b Chemical added to sealed tubes and mixed at 37 °C.

In addition, results from mutagenicity assays appear to be dependent on the exposure methods and the form of chloroprene tested (e.g., newly distilled or aged). For example, Westphal et al. (1994) confirmed the importance of both vehicle and decomposition products in assessing the mutagenicity of chloroprene. They demonstrated that freshly distilled chloroprene was not mutagenic, but chloroprene aged for as little as two to three days at room temperature was mutagenic in *S. typhimurium* TA100. The mutagenicity increased linearly with the age of the distillate, probably due to the presence of decomposition products such as cyclic dimers (Westphal et al., 1994).

Drevon and Kuroki (1979) were not able to induce point mutations when chloroprene was tested in Chinese hamster V79 cells. Himmelstein, Gladnick, Donner, Snyder, and Valentine (2001) tested the primary metabolite of chloroprene, (1‐chloroethenyl) oxirane (CEO), and found it to be mutagenic in the absence of S9, suggesting that this metabolite may be the reactive agent in the Ames tests; however, CEO was not found to be genotoxic in mammalian cells *in vitro* (Chinese hamster V79 cells) (Himmelstein, Gladnick, et al., 2001). Westphal et al. (1994) also found that addition of glutathione to the chloroprene/metabolite Ames tests significantly diminished the reported mutagenic activity. This suggests that glutathione and possibly other detoxification agents, which may not present in S9 microsome preparations at levels present in intact cells in animals, could mitigate or eliminate the production of potentially active metabolites *in vivo*.

Additional evidence challenging the mutagenicity of chloroprene is the distinct mutagenic profile of chloroprene compared to 1,3‐butadiene and isoprene—structurally similar compounds, which are known to be metabolized to an epoxide intermediate and are rodent carcinogens. While these compounds may be carcinogenic in rodents, evidence is available that shows that the mutagenic and clastogenic profiles of 1,3‐butadiene and isoprene are considerably different from the chloroprene profile (Tice, 1988; Tice et al., 1988). While 1,3‐butadiene appears to be an effective somatic cell genotoxin in mice (Table VII), chloroprene was not reported to be genotoxic in mice (Tice, 1988; Tice et al., 1988). Similar to the results reported in Tice (1988), and unlike isoprene, chloroprene was reported to have no significant changes in any cytogenetic measure of genotoxic damage, alteration in frequency of peripheral blood or in the bone marrow following administration of up to 80 ppm chloroprene six hours per day for 12 days (Tice et al., 1988). Shelby (1990) also reported negative results for chloroprene for the induction for sister chromatid exchange and micronuclei in mouse bone marrow cells following inhalation exposure, unlike 1,3‐butadiene and isoprene.

**Table VII:** Comparison of the Mutagenic Profiles of Chloroprene, 1,3‐Butadiene, and Isoprene

		*In Vivo* (B6C3F1 mouse)^a^		
Chemical	*In Vitro* Ames	CA	SCE	Micronuclei
1,3‐Butadiene	+	+	+	+
Chloroprene	+/–	–	–	–
Isoprene^b^	–	–	+	+

CA, chromosomal aberrations; SCE, sister chromatid exchanges.

a Exposure was 10–12 days (6 h/day) via inhalation (Tice et al., 1988).

b 2‐Methyl‐1,2,3,4‐diepoxybutane metabolite is positive (Gervasi & Longo, 1990).

Although the reactive metabolite of chloroprene, CEO, does induce mutations *in vitro* in bacterial strains (Himmelstein, Gladnick, et al., 2001), neither the administration of chloroprene nor the reactive epoxide metabolite was genotoxic or mutagenic *in vitro* in mammalian cells, including Chinese hamster V79 cells (Drevon & Kuroki, 1979; Himmelstein, Gladnick, et al., 2001). The absence of genotoxicity in intact mammalian cells systems and *in vivo* studies suggests that the bacterial mutagenicity data may have limited relevance to the genotoxicity of chloroprene in humans, and while *in vitro* studies do suggest an interaction between CEO and DNA adducts, this effect has not been observed *in vivo* in mammalian cells.

The only published chloroprene‐related study showing positive chromosomal aberrations *in vivo* was a study by Sanotskii (1976). This study is limited, however, because it lacked specific details on the laboratory experiments including the use of controls and experimental conditions. In addition, the authors tested only very small numbers of animals per group (<10). These results are also inconsistent with the results from more reliable and more recent studies conducted by NTP in mice (NTP, 1998; Shelby, 1990).

Two other major differences between chloroprene and 1,3‐butadiene are evident from the experimental data. First, the *ras* profile for chloroprene in lung tumors in treated animals is considerably different from that of 1,3‐butadiene (Sills, Hong, Melnick, Boorman, & Devereux, 1999). Second, the toxic effects and histopathology observed in chloroprene‐treated F344 rats and B6C3F1 mice are different from those seen in 1,3‐butadiene‐exposed animals (Melnick et al., 1996).

As part of the current literature searching performed for this assessment, three recent mechanistic studies were identified (Guo & Xing, 2016; Thomas et al., 2013; Wadugu et al., 2010). Wadugu et al. (2010) examined the potential DNA cross‐linking of CEO using a denaturing polyacrylamide gel electrophoresis to monitor possible formation of interstrand cross‐links compared to other structurally similar DNA cross‐linkers, including diepoxybutane (DEB) and epichlorohydrin (ECH), to better understand the cellular mechanisms associated with chloroprene toxicity. The authors determined that CEO forms cross‐links with deoxyguanosine residues at 5‐GC and 5‐GGC sites with the rate of cross‐link formation dependent on the pH (no cross‐links were observed at pH 7). They also reported a correlation between CEO cross‐linking formation and cytotoxicity, measured by cell survival curves from the Trypan blue exclusion assay (Wadugu et al., 2010). However, the assays in this study were conducted with isolated DNA in buffer or in isolated chicken erythro‐progenitor cells and the relevance of these results to the *in vivo* situation or to humans is unclear.

Guo and Xing (2016) and Thomas et al. (2013) conducted studies to evaluate gene expression changes in the lung of mice (Guo & Xing, 2016; Thomas et al., 2013) or rats (Thomas et al., 2013) following inhalation exposure to chloroprene for five or 15 days at a range of concentrations (0.3–200 ppm) that covers the concentrations administered to each species in the NTP (1998) study. In the Thomas et al. (2013) study, female mice were exposed through whole‐body inhalation to 0 ppm, 0.3 ppm, 3 ppm, 13 ppm, and 90 ppm chloroprene and female rats were exposed similarly to 0 ppm, 5 ppm, 30 ppm, 90 ppm, or 200 ppm of chloroprene six hours per day, five days per week for five or 15 days. Following necropsy, a histological examination was performed, and no treatment‐related effects were reported in rats and the only effect reported in mice was minimal epithelial hyperplasia of the terminal bronchioles exposed to 90 ppm for five or 15 days. Gene expression changes were analyzed using an ANOVA approach with a pathway enrichment analysis and a benchmark dose (BMD) analysis, focusing on pathways with a dose–response that was consistent with the cross‐species differences in lung tumor incidence. In the enrichment analysis, 18 pathways were enriched in the mouse lung at concentrations ≥12.8 ppm and only one of the same pathways was enriched in the rat lung at concentrations ≤80 ppm. For the pathway‐based BMD analysis, 11 pathways had median BMD values similar to the BMD value for lung tumors in the mouse. However, when these pathways were filtered to remove those with a median BMD value in the rat of less than 80 ppm, only four pathways remained. When considering the results of both analyses, only two pathways overlapped and these included glutathione metabolism and methionine–cysteine–glutamate metabolism. Therefore, the results are consistent with the generation of reactive metabolites as a key event in the MOA for chloroprene‐inducted lung tumors in rodents. However, while Thomas et al. (2013) note that bioinformatic tools have continued to improve the interpretation of gene expression microarray, there are still some challenges when using gene expression data to identify a MOA for a chemical, including nonspecific effects of chemicals outside of the primary molecular mechanism, adaptive cellular responses and temporal differences in phenotypic responses and gene expression changes.

Guo and Xing (2016) obtained the transcription profiles submitted by Thomas et al. (2013) to the Gene Expression Omnibus and conducted a reanalysis of the gene expression data in the mouse lung, dividing the results into noncarcinogenic exposures (0.3 ppm and 2 ppm) and carcinogenic exposures (13 ppm and 90 ppm). Twelve gene modules with multiple biological activities out of 2,434 differentially expressed genes were reported by the study authors to be associated with the lung carcinogenesis of chloroprene. The authors concluded the results suggest that lung cancer induced by chloroprene is a complex and multistep process that involves multiple biological activities including protein processing, nutrition metabolism, immune response, signal transduction and RNA metabolism, modification and DNA repair. They also noted seven “Hub” genes, two of which were upregulated and involve phase II detoxification and aldehyde‐containing elimination, with the remaining hub genes downregulated.

### 3.3 Chloroprene Toxicity Mode of Action (MOA)

It is hypothesized that chloroprene is metabolized to epoxides (primarily CEO) via cytochromes P4502E1 (CYP2E1), and that these epoxides are the toxic moiety responsible for the observed chloroprene toxicity, including the development of tumors in animals. In the 2010 Review, EPA concluded that chloroprene was mutagenic and acted via a mutagenic MOA. Support for this MOA was limited to selective results from positive bacterial assays, the observation of protooncogenes in mice from the NTP (1998) study, and structural similarities with 1,3‐butadiene. Himmelstein, Carpenter, Hinderliter, Snow, and Valentine (2001) were the first to quantitatively identify CEO as the primary epoxide metabolite (via CYP2E1) of chloroprene in the liver microsomes of B6C3F1 mice, F344 and Wistar rats, and Syrian hamsters, and also humans. The formation of this epoxide was speculated to be related to the carcinogenic MOA of chloroprene similar to that of 1,3‐butadiene. However, there is also evidence that suggests that chloroprene might act via an alternative MOA. Consistent with recommendations by NRC (2011, 2014) for evidence integration and WOE analyses, and as recommended by EPA (2005a) Cancer Risk Assessment guidelines, we evaluated the plausibility of an alternative MOA for chloroprene.

Evidence suggests an alternative MOA for chloroprene could be one involving chronic, cytotoxicity‐driven hyperplasia. At high concentrations, chloroprene was clearly toxic to animals, resulting in a high death rate, but did not demonstrate *in vivo* genotoxicity (Shelby, 1990), supporting a MOA based on target‐site cytotoxicity. In mice, histopathological evaluations of target tissues following chloroprene exposures are consistent with a nongenotoxic MOA. For example, the incidence of chloroprene‐induced bronchiolar hyperplasia in the respiratory system at elevated chloroprene exposures follows the increased incidence of lung tumors in a dose‐dependent manner, whereas the incidence of lung K‐*ras* mutations (a precursor of many cancers) does not. As described by Sills et al. (1999), the K‐*ras* transversions exhibited a reverse dose–response relationship with greater K‐*ras* transversions in the lowest exposure group of 12.8 ppm (8/10), compared with the highest exposure group of 80 ppm (4/22). Also, Melnick et al. (1996) reported that the toxicity and histopathology observed in chloroprene‐treated F344 rats and B6C3F1 mice were consistent with a cytotoxicity‐driven hyperplasia, which can result from cell injury or death and subsequent tissue regeneration. Buzard (1996) hypothesized that hyperplastic processes lead to selection of pre‐existing oncogene and tumor suppressor gene mutations. A similar MOA has been proposed for naphthalene, which is also cytotoxic at tumorigenic exposures but has equivocal evidence of genotoxicity (Bogen et al., 2008).

### 3.4 Kinetics of Chloroprene Metabolism

In addition to the mechanistic studies that have sought to establish the mutagenicity of chloroprene and a likely MOA, several toxicokinetic studies have been conducted to characterize the metabolism of chloroprene *in vitro* in lung and liver tissue fractions of rats, mice, hamsters, and humans (Himmelstein, Carpenter, Evans, Hinderliter, & Kenyon, 2004; Himmelstein, Carpenter, & Hinderliter, 2004; Himmelstein, Carpenter, et al., 2001). Chloroprene is proposed to initially undergo oxidation by cytochrome P450 (CYP450) to form two epoxides, either CEO or 2‐chloro‐2‐ethenyloxirane. These two epoxides are then hydrolyzed by epoxide hydrolase to form either 3‐chlorobut‐3‐ene‐1,2‐diol or 1‐hydroxybut‐3‐en‐2‐one, respectively. The 1‐hydroxybut‐3‐en‐2‐one is then either cleared by glutathione or is further metabolized to 1‐hydroxybutan‐2‐one. The 2‐chloro‐2‐ethenyloxirane epoxide metabolite can undergo multiple rearrangements and form 1‐chlorobut‐3‐en‐2‐one or 2‐chlorobut‐2‐en‐1‐al, which are ultimately cleared from the body as 2‐chloro‐butanol (Cottrell, Golding, Munter, & Watson, 2001; Himmelstein, Carpenter, & Hinderliter, 2004; Himmelstein, Carpenter, et al., 2001).

As noted previously, Himmelstein, Gladnick, et al. (2001) were the first to quantitatively identify CEO as the primary epoxide metabolite (via CYP2E1) of chloroprene in the liver microsomes of B6C3F1 mice, F344 and Wistar rats, Syrian hamsters, and humans. Greater amounts of CEO measured in mice liver microsomes and liver microsomes from both species of rat, were observed compared to that measured in liver microsomes of humans and hamsters (Himmelstein, Carpenter, et al., 2001). Species differences in the hydrolysis of CEO were reported, with hydrolysis reported to occur faster in hamsters∼humans>rats>mice. Species‐specific differences were also reported in glutathione conjugation, with slower rates reported in the mouse compared to either species of rats or hamsters. The authors concluded that metabolism may help explain species differences showing a greater sensitivity for chloroprene‐induced tumorigenicity in mice, for example, compared with hamsters. Cottrell et al. (2001) reports similar findings to those of Himmelstein, Carpenter, et al. (2001) and further defines the metabolism of chloroprene and its metabolites in liver microsomes of B6C3F1 mice, Sprague–Dawley or F344 rats, and humans. Strikingly similar species differences in metabolism have been reported for the related compound, 1,3‐butadiene (Bond, Himmelstein, Seaton, Boogaard, & Medinsky, 1996).

Himmelstein, Carpenter, & Hinderliter (2004) further investigated the species‐specific kinetics of the metabolism of chloroprene *in vitro* in liver and lung tissue fractions of B6C3F1 mice, Fischer and Wistar rats, Golden Syrian hamsters, and humans. The study focused in the *in vitro* kinetics of chloroprene oxidation and subsequent metabolism of the CEO metabolite by microsomal epoxide hydrolase and cytosolic glutathione S‐transferases. In liver microsomes, oxidation of chloroprene, based on the *V*max/*Km* ratio, was faster in the mouse (224) and hamster (218) than in rats (125–146) and humans (101). In lung microsomes, the *V*max*/Km* ratio was much greater for mice (66.7) compared with other species (1.3). Hydrolysis of CEO in liver microsomes was faster in the human and hamster than for the rat or mouse, with the ratios in the liver 3 to 11 times greater than those in the lung.

### 3.5 PBPK Modeling Studies

**Figure 2:**
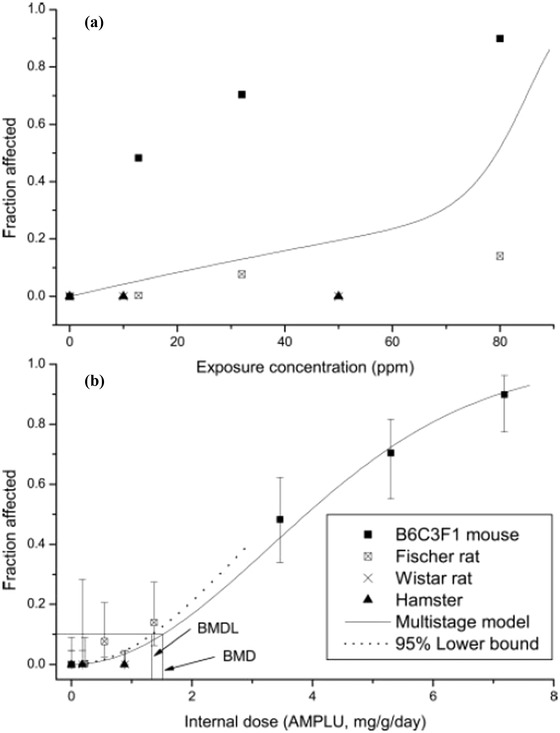
Comparison of tumor dose–responses for inhalation exposures of male mice, rats, and hamsters to chloroprene using (A) inhaled chloroprene concentration or (B) PBPK model‐predicted daily production of chloroprene epoxides per gram of lung. Based on inhaled concentration, the male mouse response is significantly higher than the other species (A); however, using the rate of chloroprene epoxide production, a similar dose–response curve is consistent with the data from all species studied.

The *in vitro* metabolism data from Himmelstein, Carpenter, & Hinderliter (2004) were used to develop a PBPK model for chloroprene in the mouse, rat, hamster, and human (Himmelstein, Carpenter, Evans, et al., 2004). The model was able to reproduce the time course for chloroprene concentrations in closed‐chamber inhalation studies conducted in Wistar and F344 rats, B6C3F1 mice, and Syrian golden hamsters. The PBPK model was then employed to estimate the relevant internal dose metrics (daily production of chloroprene epoxides per gram of lung) to support an interspecies comparison of lung tumor risk (Himmelstein, Carpenter, Evans, et al., 2004). Strikingly, whereas the dose–responses for tumors in mice, rats, and hamsters differed widely when chloroprene exposure concentration was used as the metric (Fig. 2A), a consistent dose–response relationship across all species was observed when the PBPK dose metric was used (Fig. 2B). The ability of the model to adjust for the impact of species differences in metabolism on cancer risk from chloroprene exposure suggests that its use to support animal‐to‐human extrapolation would be critical to any risk assessment. Interestingly, while there is consistency of the tumor incidence across male animals of different species and strains, female mice exhibit a higher tumor incidence than male mice at the same rate of lung metabolism. Few studies have explored gender differences in the responses to chemical exposures. One study, that evaluated the toxicity of permethrin, however, shows that there is a proliferative response of Club cells in the female mouse lung that is not observed in the male mouse lung (Yamada et al., 2017). In addition, studies of naphthalene lung toxicity have demonstrated a greater sensitivity of the female mouse lung to both acute and repeated exposures (Sutherland, Edwards, Combs, & Van Winkle, 2012; Van Winkle, Gunderson, Shimizu, Baker, & Brown, 2002). Therefore, it is likely that the greater susceptibility to a proliferative response in the female mouse lung results from gender differences in the tissue response to damage rather than metabolism differences.

A subsequent study (Yang et al., 2012) provided *in vitro* data on metabolism in the kidney in order to extend the applicability of the Himmelstein, Carpenter, Evans, et al. (2004) chloroprene PBPK model to risk assessments for renal tumors, and Markov chain Monte Carlo analysis was conducted to estimate the uncertainty in all of the *in vitro* metabolism parameters (liver, lung, and kidney) used in the PBPK model. The model was also used to calculate lung, liver, and kidney metabolism dose metrics in mouse, rat, and human under the animal bioassay exposure conditions (six‐hour inhalation exposures at 12.8 ppm, 32 ppm, and 80 ppm). The chloroprene PBPK model was also used in a study comparing animal‐ and human‐based estimates of lung cancer risk from chloroprene (Allen et al., 2014), which will be discussed later (Section 5). As part of this study, the PBPK model from Yang et al. (2012) was used to calculate the lung dose metric for a human occupational exposure to chloroprene at 1 ppm; this human dose metric, together with the animal bioassay dose metrics from Yang et al. (2012) was used in the present analysis to estimate an updated IUR for chloroprene.

In a follow‐up publication, the development of a fully validated PBPK model for chloroprene in the male and female mouse, rat, and human based on *in vitro* studies is described and the model is used to calculate an updated cancer unit risk factor (Clewell et al., 2019). The analyses include multiple sensitivity analyses to address uncertainties in the data and methods.

## 4 EPIDEMIOLOGICAL EVIDENCE

In this section, we evaluate the epidemiological evidence of chloroprene carcinogenicity. The epidemiological literature on chloroprene exposure and cancer risk includes studies of occupational cohorts from several countries published over 30 years (Bulbulyan et al., 1998; Bulbulyan et al., 1999; Colonna & Laydevant, 2001; Leet & Selevan, 1982; Li, Dong, Liu, & Liu, 1989; Marsh et al., 2007a, 2007b; Pell, 1978). The early results from the Pell (1978) and Leet and Selevan (1982) studies were included in the most recent update by Marsh et al. (2007a, 2007b). Chinese (Li et al., 1989), Russian (Bulbulyan et al., 1998), and Armenian (Bulbulyan et al., 1999) studies have not been updated and have serious limitations that have been discussed elsewhere (Acquavella & Leonard, 2001; Bukowski, 2009; Rice & Boffetta, 2001) and summarized briefly below.

The study by Li et al. (1989) included 1,213 workers exposed to chloroprene for at least one year starting in 1969 and followed until 1983 (14 years). For the total cohort, statistically significant standardized mortality ratios (SMRs) were reported for all cancers combined (SMR = 2.38) and for a category called “other sites” (consisting of one pancreatic, one ocular, and one tonsillar cancer); however, only 16 deaths due to any cancer were observed leading to statistical instability and possibly false‐positive findings. SMRs were calculated using only three years (1973–1975) of local cancer mortality rates, and this short duration may not be representative of the entire study period. Furthermore, for liver and lung cancers, the authors were unable to control for important potential confounders such as chronic infection with hepatitis B virus and aflatoxin B1, alcohol consumption, or tobacco smoking (Chen et al., 2003; Lee et al., 2009; Stuver & Trichopoulos, 2008).

Bulbulyan et al. (1998) evaluated chloroprene exposure and cancer mortality among 5,185 shoe factory workers employed from 1979 to 1993 in Moscow, Russia. Exposures were estimated based on job histories and industrial hygiene data. Increased risk and mortality for liver cancer incidence (standardized incidence ratio [SIR] = 3.27, 95% confidence interval [CI]: 1.47–7.27, *n* = 6) and mortality (SMR = 3.39, 95% CI: 1.09–10.5, *n* = 3) were reported. Similar to the Li et al. (1989) study, Bulbulyan et al. (1998) calculated expected numbers based on mortality and incidence rates for Moscow for the two years (1992 to 1993) during which liver cancer rates among women had dropped to their lowest point after peaking in the late 1970s (Levi, Lucchini, Negri, Boyle, & La Vecchia, 2004) resulting in inflated risk estimates.

Bulbulyan et al. (1999) assessed the risk of cancer incidence and mortality among 1,897 men and 417 women exposed to chloroprene in a production plant in Yerevan, Armenia, from 1940 to 1988. Reduced incidence of all cancers combined (SIR = 0.68, 95% CI: 0.49–0.94, *n* = 37) and cancer mortality (SMR = 0.87, 95% CI: 0.56–1.36, *n* = 20) were reported. However, liver cancer incidence was increased (SIR = 3.27, 95% CI: 1.47–7.27, *n* = 6) and lung cancer incidence was decreased (SIR = 0.53, 95% CI: 0.24–1.19, *n* = 6) among workers with the highest and longest duration of exposure. Similar findings were reported for cancer mortality. Primary limitations included the lack of information on important potential confounding factors and failing to employ local reference rates in deriving SIRs and SMRs.

In the populations represented in both the Russian and Armenian cohorts, there is a high incidence of alcoholic cirrhosis, a well‐known risk factor for liver cancer (London & McGlynn, 2006): 11 deaths from cirrhosis of the liver (three in males and eight in females) in the Russian cohort (Bulbulyan et al., 1998) and 32 cases (27 in males and five in females) in the Armenian cohort (Bulbulyan et al., 1999). Also, none of these studies evaluated potential confounding by smoking. Furthermore, the interpretation of SIR and SMR estimates is challenging when they are based on very small expected values, resulting in highly unstable relative risk (RR) estimates and false positive (or negative) results (Checkoway, Pearce, & Kriebel, 2004).

In contrast with the studies discussed above, Marsh et al. (2007a, 2007b) is the largest and methodologically strongest epidemiology study of chloroprene‐exposed workers. The cohort included 12,430 workers ever employed at plants producing and using chloroprene in Louisville, KY (*n* = 5,507); Pontchartrain, LA (*n* = 1,357); Maydown, Northern Ireland (*n* = 4,849), or Grenoble, France (*n* = 717). SMRs (using national and regional standard populations) were calculated stratified by selected demographic, work history and exposure factors, and used worker pay type (White or Blue collar) as a rough surrogate for lifestyle factors including smoking. An exposure reconstruction assessment was conducted based on mathematical modeling of potential exposures for each process and work category. Detailed work histories were used to link individual cohort members to exposure estimates and these were summed to estimate cumulative exposure.

Overall, and for all subcohorts defined by specific plant(s) in Marsh et al. (2007a, 2007b), SMRs based on local reference rates were all below 1.0, providing no indication of any excess cancers among chloroprene‐exposed workers. We conducted power calculations based on exact Poisson probabilities, at alpha = 0.5 (one‐sided) to detect an excess. For lung cancers, the study had overall nearly 100% power to detect a true RR of 1.5, and over 95% power (96–100%) to detect a true RR of 1.5 in each of the exposure subcategories except the middle cumulative exposure group, for which the power was 82%. As liver cancer is much rarer, the study overall had 61% power to detect a true RR of 1.5 and 97% power to detect a true RR of 2.0. For about half of the exposure subcategories, the power was 80% or higher to detect a true RR of 2.5 and for all but one (the middle cumulative exposure group), the power was at least 84% to detect a true RR of 3.0.

**Figure 3:**
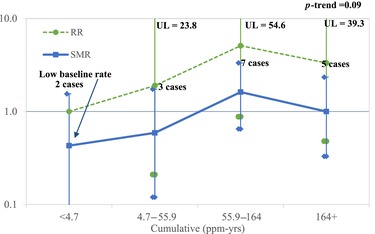
Liver cancer RRs and SMRs by cumulative chloroprene exposure, Louisville, KY.

Despite the lack of any excess occurrence of liver cancers, Marsh et al. (2007b) calculated RRs by category of exposure, using the lowest exposure category as the referent. However, this category exhibited a deficit of liver cancers (six observed, about 10 expected) and, therefore, was not representative of liver cancer rates among unexposed persons. When rates of liver cancer for higher exposure categories (none of which exhibited an excess relative to expected) were divided by the anomalously low reference rate, the resulting RRs were artificially inflated by 39%, 38%, and 57%, respectively. As shown in Table VIII and Fig. 3, the RRs are simply a reflection of the SMRs, inflated by the exact amount of the deficit in the referent group. Because of this deficit, the comparison group used was not appropriate and the resulting RRs are misleading. As shown by the SMR calculations, there was no excess of liver cancer among the chloroprene‐exposed workers. In fact, there was a slight overall deficit of liver cancer. A similar issue has been observed in the evaluation of associations between formaldehyde exposures and leukemia mortality (Checkoway et al., 2015).

**Table VIII:** Exposure‐Response Analysis for Chloroprene and Liver Cancers, Based on Relative Risk (internal referent) and SMR Analysis (external referent) Estimates, Louisville Plant

		Relative Risk Analysis			SMR Analysis	
Liver Cancer	Deaths	#Cases	RR (95% CI)	*p*‐value	Person‐years	SMR (95% CI)
Exposure duration (years)						
<10	6	1,500	1.00	Global = 0.24	1,31,276	0.61 (0.22–1.32)
10–19	4	216	3.85 (0.75–7.09)	Trend = 0.36	30,404	2.08 (0.57–5.33)
20+	7	965	1.75 (0.49–6.44)		36,239	0.99 (0.40–2.04)
Average Intensity of Exposure (ppm)						
<3.62	3	714	1.00	Global = 0.22	69,274	0.62 (0.13–1.80)
3.62–8.12	7	568	3.81 (0.77–5.76)	Trend = 0.84	27,933	1.73 (0.70–3.56)
8.12–15.99	3	388	1.84 (0.22–5.74)		28,689	0.94 (0.19–2.74)
16.0+	4	1,011	1.31 (0.20–0.07)		72,023	0.59 (0.16–1.52)
Cumulative exposure (ppm‐years)						
<4.75	2	744	1.00	Global = 0.17	68,918	0.43 (0.05–1.55)
4.75–55.19	3	725	1.9 (0.21–23.81)	Trend = 0.09	56,737	0.59 (0.12–1.74)
55.91–164.0	7	653	5.1 (0.88–54.64)		39,840	1.62 (0.65–3.33)
164.0+	5	559	3.33 (0.48–9.26)		32,424	1.00 (0.33–2.34)

Source: Marsh et al. (2007b), Table IV.

RR, relative risk; CI, confidence interval; ppm, parts per million; SMR, standardized mortality ratio.

The chloroprene‐exposed workers in Marsh et al. (2007a, 2007b) had only about 90% of the expected mortality rate overall (17 observed with about 19 expected), based on a nonexposed population reference rate. Our interpretation of Marsh et al. (2007a, 2007b), consistent with the authors’, is that the evidence does not demonstrate an association between occupational chloroprene exposure and incidence of human cancers.

A fundamental part of evidence integration is weighing the evidence. Confidence in results depends substantially on study quality. Bukowski (2009) evaluated the quality of the occupational epidemiological studies of chloroprene‐exposed workers using an approach consistent with EPA (2005a) and the subsequent NRC (2014) recommendations. The evaluation considered the eight mortality studies of seven chloroprene‐exposed cohorts from six countries including the Chinese, Armenian, Russian, and US/European studies (Table IX). Each study was assigned to categories of high, medium, or low quality for each of 10 quality criteria and an overall WOE assessment was performed. The four‐cohort pooled study (Marsh et al., 2007a, 2007b) was found to be the most methodologically rigorous epidemiology study (Table IX). For example, as shown in Table X, the Marsh et al. (2007a, 2007b) studies had a far greater number of subjects than other occupational studies. Also, it had the most comprehensive exposure assessment including assessment of potential confounders.

**Table IX:** Quality Rankings for Cohort Studies of Cancer Risks from Occupational Chloroprene Exposure

	Marsh et al.’s Study				Other Studies			
US EPA Criteria	Kentucky^a^	North Ireland^a^	Louisiana^a^	France‐Mort* ^,^ a	Armenia^b^	France‐Incid** ^,^ c	Russia^d^	China^e^
Clear objectives	H‡	H	H	H	H	H‐M	H	M
Comparison groups	H	H‐M	H‐M	M	M	M	M‐L	L
Exposure	H	H	H	H	M	M	L	L
Follow‐up	H	H‐M	H	H‐M	M‐L	M‐L	M‐L	M‐L
Case ascertainment	H	H‐M	H‐M	H‐M	M	M	M	H‐M
Control of bias	H‐M	H‐M	H‐M	M	M‐L	M	M	M‐L
Sample size	H	H	M	L	M‐L	L	H‐M	M‐L
Data collection and evaluation	H	H	H	H	M	M	M‐L	M‐L
Adequate response	H	H	H	H	M	M	M	H‐M
Documentation of results	H	H	H	H	M‐L	M	M	L
**Overall rank (1 = best)**	**1**	**2**	**3**	**4**	**5**	**5**	**5**	**6**

Source: Bukowski (2009).

^*^Mort = Mortality.

^**^Incid = Incidence.

^‡^Subjective estimate of study quality for each specific criterion H = high, M = medium, L = low.

a Marsh et al. (2007a, 2007b).

b Bulbulyan et al. (1999).

c Colonna and Laydevant (2001).

d Bulbulyan et al. (1998).

e Li et al. (1989).

**Table X:** Relative Size of Marsh et al. (2007a, 2007b) Study Compared with Other Studies

Study	Subjects (Person‐years)	Lung Cancer Deaths	Liver Cancer Deaths
Bulbulyan et al. (1998)	5,185 (70,328)	31	10
Bulbulyan et al. (1999)	2,314 (21,107)	3	3
Colonna and Laydevant (2001)	717 (17,057)	9	1
Leet and Selevan (1982)	This study cohort was included in Marsh et al. (2007a, 2007b)		
Li et al. (1989)	1,258 (20,105)	2	6
**Total Other Studies**	**9,474 (128,597)**	**45**	**20**
Marsh et al. (2007a, 2007b) (L)	5,507 (197,010)	266	17
Marsh et al. (2007a, 2007b) (M)	4,849 (127,036)	48	1
Marsh et al. (2007a, 2007b) (P)	1,357 (30,660)	12	0
Marsh et al. (2007a, 2007b) (G)	717 (17,057)	10	1
**Total** Marsh et al. (2007a, 2007b)	**12,430 (372,672)**	**336**	**19**
**Combined Studies**	**21,904 (501,269)**	**381**	**39**
Marsh et al. (2007a, 2007b **) (all)**	**57 (74%)**	**88%**	**49%**

L, Louisville, Kentucky; P, Ponchatrain, Louisiana; M, Maydown, Northern Ireland; G, Grenoble, France.

Therefore, the study by Marsh et al. (2007a, 2007b) is the only one of adequate quality to validly address epidemiologically the cancer risks associated with human exposure to chloroprene (precluding meaningful meta‐analyses).

Taken as a whole, the epidemiological evidence on chloroprene and cancer is insufficient to conclude that chloroprene causes cancer in humans, and the strongest studies demonstrate no increased risk of liver or lung cancers (or any other cancer) from occupational exposure to chloroprene.

## 5 CALCULATING THE CHLOROPRENE IUR

The strongest epidemiological studies on chloroprene‐exposed workers and cancer are negative, and therefore, do not provide adequate data for deriving an IUR for chloroprene. Therefore, we calculated an IUR based on data from animal studies and PBPK modeling from Yang et al. (2012) to adjust for the significant differences in the way humans respond to and metabolize chloroprene versus laboratory animals. In the 2010 Review, EPA similarly used the animal data to derive an IUR for chloroprene, noting that the study limitations of the epidemiological data precluded development of quantitative risk estimates, but choosing not to use a PBPK model to adjust for species differences. Prior to the EPA (2010) Review, the agency had concluded that the published PBPK model (Himmelstein, Carpenter, Evans, et al., 2004) lacked adequate validation, because the only *in vivo* studies that had been conducted to support the PBPK model development were closed‐chamber inhalation studies in which the animals’ ventilation rate had not been measured. The agency believed that without a measured ventilation rate, the closed‐chamber data could not provide an unequivocal validation of the PBPK model. In response to the EPA criticism, a nose‐only inhalation study was conducted in which mice were exposed to chloroprene at the bioassay concentrations of 12.8 ppm, 32 ppm, and 80 ppm and the ventilation rate was measured. Although the results of this study (International Institute of Synthetic Rubber Producers [IISRP] 2009) were submitted to the EPA docket, the agency did not re‐evaluate the PBPK model before publishing the 2010 Review.

The IUR calculated in the current assessment for chloroprene was conducted using the same standard methodologies that the EPA has employed in IRIS assessments for carcinogens including dichloromethane, vinyl chloride, tetrachloroethylene, carbon tetrachloride, and acrylamide. This methodology involves using a PBPK model to estimate an internal dose at the target organ of interest (e.g., the lung), based on the MOA. The hypothesized MOA for chloroprene is that chloroprene itself does not exert a carcinogenic effect, but rather a metabolite of chloroprene is carcinogenic. Therefore, carcinogenicity depends on the internal concentration of the metabolite and not the internal (or external) concentration of chloroprene. Applying this methodology significantly reduces the uncertainty associated with extrapolating results from animal experiments to humans and reflects underlying pharmacokinetic differences across species. The EPA (2005a) “Guidelines for Carcinogen Risk Assessment” note that toxicokinetic or PBPK modeling is the preferred approach for estimating dose metrics from exposure.

When an IUR is based on animal data and a PBPK model is used, an animal PBPK model is required to estimate the internal dose corresponding to each of the administered concentrations (i.e., ppm in the chamber air), following the same pattern of exposure of the animals in the study (e.g., days/week). This internal dose estimate is then used (instead of the air concentration) for dose–response modeling when estimating a Point of Departure (POD). This POD corresponds to the internal dose in the animal. The human PBPK model then is applied to account for known physiological and metabolic differences between the animal and human. This is accomplished by estimating the equivalent external concentration that results in the internal dose equal to the POD derived from the animal data. The IUR is estimated by dividing the risk level (benchmark risk or BMR associated with the POD) by the POD. The IUR is interpreted as the risk per unit (ppm or µg/m^3^) intake.

Chloroprene PBPK modeling results for mice, rats, and humans are reported in Yang et al. (2012). Specifically, the internal dose estimates associated with the concentrations administered to both mice and rats in the NTP (1998) study are provided, including gender‐specific internal doses, that is, the average amount of chloroprene metabolized per day per gram of lung (AMPLU), based on the PBPK model simulations. These internal doses represent the concentration of the hypothesized toxic moiety (i.e., the chloroprene metabolite). The Yang et al. (2012) analysis showed that mice had the greatest amount of chloroprene metabolized per gram of lung, followed by rats and then humans. The human and rat data showed linear dose–responses over the range of NTP bioassay concentrations of 12.8 ppm, 32 ppm, and 80 ppm. Allen et al. (2014) used the PBPK model of Yang et al. (2012) to determine the relationship between the internal dose and the external exposure (ppm) in the human: they determined that 0.00352 µmole of chloroprene metabolized/g‐lung/day was calculated by model simulations conducted at either a 1 ppm occupational exposure scenario or with the adjusted continuous exposure equivalent of 0.33 ppm, supporting a relationship of 0.0106 µmole of chloroprene metabolized per gram of lung tissue per day per ppm for continuous exposure.

We relied on the internal dose results from the PBPK modeling conducted and reported by Yang et al. (2012) and Allen et al. (2014), consistent with the PBPK modeling approach that EPA has used in other IRIS assessments, to conduct the dose–response assessment. In addition, consistent with the NTP (1998) results regarding the most sensitive endpoint in the most sensitive species, we estimated the chloroprene IUR using the results for the combined incidence of alveolar/bronchiolar adenomas and carcinomas (the most sensitive endpoint) in female mice (the most sensitive species and gender).

Using the internal doses for female mice from Yang et al. (2012) (Table XI), time‐to‐tumor modeling of the incidence of lung alveolar/bronchiolar adenomas and carcinomas was performed using the multistage Weibull model provided with the EPA BMDS software (February 25, 2010 version). The multistage Weibull model has the following form:

$$\begin{array}{ccc} P\left(d,t\right) & = & 1-\exp\left[-\left(b_{0}+b_{1}d+b_{2}d^{2}+\cdots +b_{k}d^{k}\right)\right.\\ & & \left.\times \;{\left(t-t_{0}\right)}^{c}\right], \end{array}$$

where *P*(*d*,*t*) represents the lifetime risk (probability) of cancer at dose *d* (the human equivalent exposure in this case) at time *t* (a human lifetime in this case); parameters *b_i_* ≥ 0, for *I* = 0, 1, …, k; *t* is the time at which the animal's tumor status, either no tumor, tumor, or unknown (missing or autolyzed) was observed; *t*_0_ is the latency of response; and *c* is a parameter that characterizes the change in response with age. The latency (*t*_0_) was set to zero for all models. The power term parameter *c* normally is a parameter estimated by the BMD software.

**Table XI:** Internal and External Dose Metrics for Chloroprene

	PBPK Internal Dose Metric^a^
	(µmole CD metabolized/g‐lung tissue/day)
External Dose (ppm)	Mouse
12.8	0.74
32	1.19
80	1.58

a Data from Yang et al. (2012), Table V.

Time‐to‐tumor dose–response modeling is preferred and was used in the 2010 Review to model the incidence of tumors from the NTP (1998) bioassay. This was necessary, as the survival of the female mice exposed to chloroprene was “significantly less than that of the chamber control” (NTP, 1998). Time‐to‐tumor models adjust for early death of the animal, and thus, the probability that the animal, if it had lived longer, may have developed the tumor of interest.

The female mouse data that were used and the modeling output are provided in the Supporting Information. The alveolar/bronchiolar adenomas or carcinomas were all considered to be incidental tumors, consistent with the time‐to‐tumor dose–response models and approaches used by EPA in the 2010 Review. One tumor was classified as unknown in one animal in the 12.8 ppm group, so modeling was conducted both including and excluding that animal to determine if there was any major impact on the outcome of the dose–response modeling.

Consistent with the EPA approach in the 2010 Review, we selected a BMR of 1% (see Supporting Information for multistage Weibull modeling results). Note that models including or excluding the animal for which occurrence of the tumor could not be determined (e.g., an unknown tumor context) (Animal #320, from the NTP study) generated the same estimated IUR. We calculated the external human dose (in ppm) by dividing the POD or lower bound on the BMD (BMDL) expressed in the internal dose metric by the factor of 0.0106 to obtain the external concentration for continuous exposure in the human in ppm associated with the internal POD. This human‐equivalent dose in ppm was obtained by dividing the BMDL by the conversion factor derived by Allen et al. (2014) (1 ppm = 0.0106 µmole/g‐ lung tissue/day). We then calculated the IUR by dividing the BMR by the human equivalent POD/BMDL in either ppm or µg/m^3^:

IUR=BMR/POD.

The final results are presented in Table XII. Using the standard EPA methods and available published data, the IUR for chloroprene was 1.4 × 10^−2^ per ppm or 4.2 × 10^−6^ per µg/m^3^. This result incorporates the results from PBPK models and adjustments necessary to extrapolate the findings from animal studies to relevant human exposure considering the differences in pharmacokinetics.

**Table XII:** Calculation of IURs

	BMR = 0.01				
Results from 2‐stage Multistage Weibull Time‐to‐tumor model	BMDL (µmole/gram lung tissue/day)	External Concentration (ppm)^a^	IUR (per ppm)	External Concentration (µg/m^3^)^b^	IUR (per µg/m^3^)
Female Mouse Lung—incidental. Animal with unknown status excluded	0.0069	0.651	0.0151	2356	4.2 × 10^−6^
Female Mouse Lung—incidental. Animal with unknown status included	0.0070	0.660	0.0152	2389	4.2 × 10^−6^

a Human doses in ppm are obtained by dividing the BMDL by the conversion factor from Allen et al. (2014) of 0.0106 µmole/gram lung tissue/day for 1 ppm of continuous exposure.

b 1 ppm = 3.62 mg/m^3^ (EPA, 2010).

## 6 COMPARISON OF THE CURRENTLY DERIVED IUR TO EPA'S 2010 IUR

In the 2010 Review, the EPA relied on the findings of the two‐year inhalation study conducted by the NTP (1998) in B6C3F1 mice and F344/N rats and selected the results for the female mouse (the most sensitive species) as the basis for deriving the chloroprene IUR. However, instead of using a PBPK model for animal‐to‐human extrapolation, the EPA used the default approach of external air concentration and applied a number of additional conservative assumptions, rounding up several interim values, and adjusting for early‐life sensitivity in deriving the final chloroprene IUR value.

Consistent with our approach, the EPA determined the POD using dose–response modeling to derive the IUR, including estimating the effective dose at a BMD concentration associated with a 1% risk level [BMD_01_] and its lower‐bound based on the lower 95% CI of the BMD_01_ (BMDL_01_) for each chloroprene‐induced tumor type in mice. Having determined that chloroprene was more potent in inducing tumors in mice than in rats, the EPA did not further consider the rat data.

In the 2010 Review, the EPA modeled each mouse tumor endpoint reported by NTP (1998) separately using the EPA multistage Weibull time‐to‐tumor model. As with our approach, the EPA conservatively considered all tumor types, both benign and malignant, and assumed that the dose–response was linear in the low‐dose range.

The critical difference in the EPA's approach compared to our approach is that the EPA did not use a PBPK model for chloroprene to adjust for differences across species, even though the EPA acknowledged that a PBPK correction would be appropriate and that a PBPK model was available (i.e., Himmelstein, Carpenter, & Hinderliter, 2004; Himmelstein, Carpenter, Evans, et al., 2004). Instead, the EPA used the default approach of applying a dosimetry adjustment factor (DAF) that accounts for some differences in the blood: air partitioning in animals compared to humans. The EPA used a DAF of 1.0 (essentially assuming equivalence of mice and humans), based on the assumption that all the lung tumors observed were the result of systemic effects from chloroprene exposures. However, there is little evidence to support the assumption that tumors in the lungs of mice are the result of systemic effects, rather than a portal‐of‐entry effect that would result from direct contact of chloroprene with lung tissue. As noted by the EPA in the 2010 Review “treating lung tumors as systemic effects returns the highest composite unit risk (approximately 60% greater) than if lung tumors are treated as portal‐of‐entry effects.”

**Figure 4:**
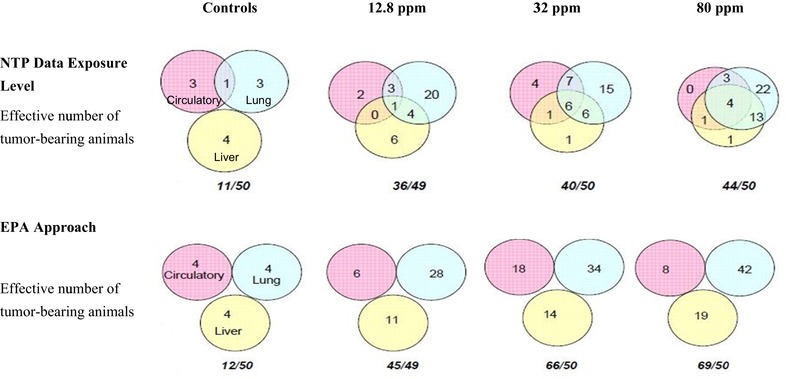
Illustration of how the EPA's approach of summing individual tumor potencies overestimates total tumor potency in female mice by assuming independence.

Although the lung was identified as the most sensitive organ in the mouse, the EPA developed a composite IUR value based on the results of dose–response modeling using the incidence of multiple tumor types in their derivation of the IUR and then estimating an upper bound on the composite risks from all tumors considered. This not only contradicts the standard approach of using the most sensitive organ in the most sensitive species, it also assumes statistical independence of different tumor types. However, the mechanism of action in multiple tissues also could be due to dependent events; for example, a liver tumor could be dependent on the generation of the same metabolite as that needed for the development of a lung tumor. Fig. 4 illustrates how the EPA's assumption of adding risk across multiple tumor sites overestimates the potential overall cancer risk. Fig. 4 also highlights the considerable nonrandom distribution of tumors in the animals bearing multiple tumors. In the 2010 Review, the EPA recognized that the assumption of independence was not verified for chloroprene, and that if this assumption did not hold, it would overestimate risk, in this case by about 50%.

As noted in the 2010 Review, the EPA assumed the independence of the observed tumors for the chloroprene‐specific data and did not evaluate the statistical independence of the multiple tumors. However, the chloroprene‐specific tumor data from the NTP (1998) study are available, specifically the tumor incidence data that the EPA relied on in deriving the IUR. We used these data to evaluate the EPA's assumption of independence using a tetrachoric correlation estimation approach (Drasgow, 1986; Olsson, 1979; Olsson, Drasgow, & Dorans, 1982; SAS, 2016). This approach is applicable when both observed variables are dichotomous, as is the case for tumor incidence data. A tetrachoric correlation assumes an underlying latent variable is affecting the observation of tumorigenic responses and that latent variable is considered to be normally distributed within an organ. Therefore, the tetrachoric estimation approach is suitable for determining if nonzero correlations exist for the incidence of tumors across sites of carcinogenicity.

We performed correlation analysis to calculate tetrachoric correlation estimates between the occurrence of a lung alveolar/bronchiolar adenoma and/or carcinoma in female mice versus the other eight tumor types used by the 2010 Review to develop a composite IUR (EPA, 2010). Table XIII shows that for most of the tumors considered by the EPA in the analysis of NTP (1998) female mice data, there was a statistically significant (*p*‐value < 0.05) correlation with the occurrence of a lung alveolar/bronchiolar adenoma and/or carcinoma with correlations of 0.25 or higher. The results from this evaluation demonstrate that the tumor incidences in the female mice in the NTP (1998) study are not statistically independent of the lung tumors, with tumors other than lung tumors occurring frequently in the same animals as those that developed lung tumors.

**Table XIII:** Correlation of Tumor Types in Female Mice from the NTP (1998) Study

Tumor Type Tested for Correlation to Lung Alveolar/Bronchiolar Adenoma and/or Carcinoma	Correlation Coefficient^a^	*p*‐value^b^	#With Lung Tumors/Total #With Tumor Type in Column 1
All organs: Hemangiosarcomas and/or hemangiomas	0.269	0.0374	25/36
Mammary gland: Carcinoma and/or adenoacanthoma	0.301	0.0242	23/32
Forestomach: Squamous cell papilloma and/or carcinoma	0.999	0.0123	5/5
Liver: Hepatocellular adenoma and/or carcinoma	0.286	0.0091	61/96
Skin: Sarcoma	0.382	0.0023	30/40
Harderian gland: Adenoma and/or carcinoma	0.131	0.3965	12/19
Zymbal gland: Carcinoma	0.957	0.0533	3/3

a Correlation coefficients greater than 0.25 indicate discernable positive correlations between the tumor types.

b *p*‐values less than 0.05 indicate that the correlations are statistically significant and the tumors are not independent.

Finally, the EPA applied an age‐dependent adjustment factor (ADAF) to account for early‐life susceptibility, assuming a mutagenic MOA. This adjustment reflects the use of several sensitivity adjustments for different life stages, which are applied for presumed mutagenic compounds as specified in EPA's “Supplemental Guidance for Assessing Susceptibility from Early‐Life Exposure to Carcinogens” (EPA, 2005b). This yielded a final adjusted unit cancer risk of 5 × 10^−4^ per µg/m^3^. Our critical evaluation of the mechanistic data indicates that there is insufficient evidence of a mutagenic MOA for chloroprene, and thus, application of the ADAF is not justified in this case. Therefore, in calculating an IUR we did not apply the ADAF.

## 7 EVIDENCE INTEGRATION

We assessed all lines of evidence on chloroprene carcinogenicity in deriving an IUR. We first evaluated the toxicological evidence. As shown in Table XIV for lung tumors, mice respond to chloroprene exposure very differently than other laboratory animals. This difference is observed for other tumor sites as well (see Tables IV and V). In addition, as described in Himmelstein, Carpenter, Evans, et al. (2004), by using the internal dose (average chloroprene metabolized per gram of lung tissue), the risk of developing tumors is shown to be much greater in B6C3F1 mice compared to Fischer rats and other species (Table XIV). This evidence not only supports large differences in response across species, but also highlights the need to consider internal dose in developing an appropriate IUR.

**Table XIV:** Exposure Dose–Response for Rodent Lung Tumors

	Exposure Concentration (ppm)	PBPK Internal Dose^a^	Lung Tumor Incidence	Number of Animals	Extra Risk (%)^b^
Hamster^c^	0	0	0	100	0
	10	0.18	0	97	0
	50	0.88	0	97	0
Wistar rat^c^	0	0	0	97	0
	10	0.18	0	13	0
	50	0.89	0	100	0
Fischer rat	0	0	3	50	0
	12.8	0.22	3	50	0.3
	32	0.55	6	49	7.7
	80	1.37	9	50	14.0
B6C3F1 mouse^d^	0	0	15	50	0
	12.8	3.46	32	50	48.3
	32	5.30	40	50	70.4
	80	7.18	46	50	89.9

a Internal dose—average daily milligrams chloroprene metabolized/g lung tissue (AMPLU).

b The incidence data were corrected for extra risk equal to (*Pi* – *Po*)/(1 – *Po*), where *P* is the probability of tumor incidence in “*i*” exposed and “*o*” control animals Himmelstein, Carpenter, Evans, et al. (2004).

c Male Syrian hamster and Wistar rat data from Trochimowicz et al. (1998).

d Male Fischer rat and B6C3F1 mouse data from Melnick et al. (1996).

As described in Section 3.2, available mechanistic evidence also supports the need to adjust for differences in pharmacokinetics across animals and between animals and humans in deriving an IUR. Specifically, Himmelstein, Carpenter, and Hinderliter (2004) and Himmelstein, Carpenter, Evans, et al. (2004) found significant differences in the metabolism of chloroprene in mice compared to other animal species and humans. The PBPK modeling conducted by Himmelstein, Carpenter, Evans et al. (2004) showed that by adjusting for differences in metabolism, higher doses of metabolized chloroprene were calculated in the lung of mice compared to other experimental animals and likely also humans. Importantly, the adjusted internal doses are consistent with the greater mouse tumor response compared to other laboratory species.

These data are also consistent with a much lower response in humans as evidenced by the negative results of the occupational studies. Applying the IUR EPA calculated in the 2010 Review at the chloroprene exposure concentrations reported by Marsh et al. (2007b), we estimated an expected number of excess cancers and compared this to the number of cancers presented by Marsh et al. (2007b). Marsh et al. (2007b) modeled the chloroprene exposures for all unique job title classes using six exposure classes for each plant over the entire period of chloroprene production in each plant. Job title classes and time‐specific chloroprene exposure estimates were linked to each worker's job history to construct a profile. These subject‐specific profiles were then used to compute the statistical estimates of worker exposures used in the risk calculations. As reported in Marsh et al. (2007b, Table 3), the cumulative chloroprene exposures for the DuPont Louisville plant were estimated to be 18.35 and 80.35 ppm‐years for the median and mean cumulative exposures, respectively (Marsh et al., 2007b). These exposure estimates were substantially greater than those estimated for other plants and, were, therefore, used in this risk evaluation as a worst‐case scenario.

To adjust the occupational exposure estimates for a lifetime of residential exposure in µg/m^3^, we followed the EPA guidelines (EPA, 2009) and adjusted the occupational exposures to account for an eight‐hour occupational exposure versus a 24‐hour residential exposure and a five‐day work per week to a seven‐day exposure period and divided the resulting cumulative exposures (ppm‐year) by 70 years. To convert the ppm to µg/m^3^, we multiplied by 3,620. To calculate the estimated excess cancers resulting from exposure to chloroprene, we then multiplied the lifetime‐adjusted residential exposure concentration by the 2010 EPA IUR, as well as our recalculated IUR (derived above), and multiplied the risk by the 5,468 exposed workers in the Louisville plant (Marsh et al., 2007b). As a worst‐case comparison, we compared the estimated excess number of cancers from chloroprene exposures to the total number of observed cancers in the cohort, that is, all of the observed cancers not any cancers that would be predicted to results from chloroprene exposure, since the Marsh et al. (2007b) analyses as described above did not find *any* excess cancers from chloroprene exposures. Table XV presents the results from this analysis.

**Table XV:** Cancer Risk Estimates Based on the EPA (2010) IUR and the Updated IUR for Chloroprene Compared with the Observed Cancer Deaths in the Louisville Plant

		Lifetime Exposure Concentration (µg/m^3^)^a^		Excess Cancers^b^		
Source	Unit risk (per µg/m^3^)	Median	Mean	Median	Mean	Total Observed Cancer Deaths^c^
EPA (2010) Multitumor, w/ADAF	5 × 10^−4^	339	1423	927	3,891	17 (liver) 266 (lung)
Recalculated IUR lung tumor	4.2 × 10^−6^			8	33	

a Cumulative exposures from Marsh et al. (2007b), adjusted for a lifetime residential exposure (ppm‐years × 10/20 m^3^ × 5/7 days/70 years* 3620 µg/m^3^ per ppm).

b Excess cancer risk calculated by multiplying the unit risk (per µg/m^3^) by the lifetime exposure (in µg/m^3^) times the total number of exposed workers in Louisville plant (5,468 workers).

c Data obtained from Marsh et al. (2007a, Table 3), and total observed lung and liver cancers (not estimated excess cancers).

This “reality check” highlights the large discrepancy between the estimated cancers that results from application of the 2010 EPA IUR and the actual observed epidemiological findings, as the 2010 EPA IUR clearly overestimates the number of expected excess cancer cases in the Louisville cohort even when compared to the total number of observed cancers. Even at the median exposure levels, the total number of estimated excess cancers exceeds the total (and not excess) overall observed number of cancer cases (*n* = 652 for all cancers), and over three times higher than the number of lung and liver cancer mortality cases (Marsh et al., 2007a). Furthermore, assuming that workers were exposed to the mean estimated chloroprene exposures for a lifetime, using the corrected IUR, the estimated number of excess cancers slightly exceeds the observed number of liver cancers and is below the observed number of lung cancers. Therefore, the updated IUR is more consistent with the epidemiological data, while remaining conservative.

This analysis demonstrates that the 2010 EPA IUR greatly overestimates risk, and that the corrected IUR, including a PBPK adjustment, provides a better fit to the best available human data.

The derived IUR is also consistent with the IUR derived by Allen et al. (2014). Allen et al. (2014) combined the results from the most recent PBPK models for chloroprene (Yang et al., 2012) with a statistical maximum‐likelihood approach to test commonality of low‐dose risk across species. Allen et al. (2014) evaluated the difference between risk estimates obtained using external (chamber air concentrations) and internal dose (calculated with the PBPK model) metrics. The authors found that for chloroprene, external concentration‐based estimates were not appropriate for calculating and comparing cancer risks across species. By accounting for the amount of chloroprene that is metabolized per gram of tissue at the target site for different species, the PBPK results provided a substantially better fit of the models to the data. Importantly, the differences in internal dose across species explained the greater response in mice as well as the lower response of humans.

Allen et al. (2014) derived cancer unit risks for respiratory system cancers using the PBPK model results from both animal and human data that ranged from 2.9 × 10^−5^ to 1.4 × 10^−2^ per ppm (8.1 × 10^−9^ to 3.9 × 10^−6^ per µg/m^3^), with a maximum‐likelihood estimate of 6.7 × 10^−3^ per ppm (1.86 × 10^−6^ per µg/m^3^). These estimates are consistent with the IUR, we calculated of 1.5 × 10^−2^ per ppm (4.2 × 10^−6^ per µg/m^3^).

The EPA IUR predicts excess cancer risk in the St. John the Baptist Parish community around the Neoprene manufacturing plant due to chloroprene exposures. We reviewed the recent reports published by the Louisiana Tumor Registry of cancer rates at the Parish and census tract level. These reports show that the cancer incidence and mortality rates for all cancers and for lung cancer are not statistically significantly different from state‐wide rates both at the Parish level and the census tract level. These data provide further evidence that chloroprene exposures are not contributing to excess cancers in the community where the Neoprene facility is located (Maniscalco et al., 2018; Maniscalco et al., 2019).

## 8 DISCUSSION AND CONCLUSIONS

A critical review, synthesis and integration of the available evidence from epidemiological, toxicological, and mechanistic studies, indicates that epidemiological studies are largely negative and, therefore, insufficient for deriving a chloroprene IUR. This is consistent with EPA's assessment in the 2010 Review. Although animal studies provide a positive response for carcinogenicity, the toxicological evidence demonstrates major differences in species‐specific cancer response to chloroprene exposure. Quantitative differences in pharmacokinetics across species, specifically related to differences in metabolism of potentially active metabolites, must be incorporated in the derivation of an IUR. In evaluating all lines of evidence and calculating a chloroprene IUR, our approach comports with EPA methods and guidance, as well as the recommendations made by multiple NRC Committees evaluating the IRIS evaluation methods. Our IUR is also similar in magnitude to that of other chemicals classified as known human carcinogens and when used to estimate cancer risks among occupationally exposed cohorts, generates a high but much more believable cancer risk prediction than the 2010 EPA IUR.

Our critical review and synthesis of the epidemiological studies of chloroprene‐exposed workers, using standard methods that consider study quality and several potential sources of bias, indicates no clear or consistent association between occupational chloroprene exposure and mortality from lung or liver cancers (or any other) at the levels of exposures experienced by workers in these studies. The strongest study, in fact, demonstrates small deficits in lung and liver cancer mortality among chloroprene‐exposed workers (Marsh et al., 2007a, 2007b). This supports the hypothesis that humans are different from and far less susceptible to the potential carcinogenicity of chloroprene than mice, due to the way humans (and other animals) metabolize chloroprene compared to mice. A “reality check” calculation comparing the epidemiological data from Marsh et al. (2007a, 2007b) to the IUR derived by EPA confirms that the EPA's IUR overestimates cancer incidence in humans exposed to chloroprene.

Using standard methods consistent with the NRC recommendations and EPA Guidelines, and based on the most current and best quality scientific evidence, we derived an IUR for chloroprene of 4.2 × 10^−6^ per µg/m^3^. This IUR is 119 times lower than that derived by EPA and is consistent with all relevant lines of evidence on chloroprene carcinogenicity. Our analysis indicates that updating EPA's 2010 IUR for chloroprene is warranted.

## References

[risa13397-bib-0001] Acquavella, J. F. , & Leonard, R. C. (2001). A review of the epidemiology of 1,3‐butadiene and chloroprene. Chemico‐Biological Interactions, 135–136, 43–52. 10.1016/s0009-2797(01)00169-7 11397380

[risa13397-bib-0002] Allen, B. C. , Van Landingham, C. , Yang, Y. , Youk, A. O. , Marsh, G. M. , Esmen, N. , & Himmelstein, M. W. (2014). A constrained maximum likelihood approach to evaluate the impact of dose metric on cancer risk assessment: Application to beta‐chloroprene. Regulatory Toxicology and Pharmacology, 70(1), 203–213. 10.1016/j.yrtph.2014.07.001 25010378

[risa13397-bib-0003] Ambartsumyan, A. K. , Khapkina, K. S. , & Agoyan, S. G. (1968). Oxidation of chloroprene. Armyanskii Khimicheskii Zhurnal (Armenian Chemical Journal), 21(4), 290–294.

[risa13397-bib-0004] Avakian, V. M. , Gasparian, Ye I. , Avetisian, N. O. , & Kondakova, I. A. (1956). Results of a three‐year study of changes in the function of certain organs and systems of chloroprene production workers. Trudy Erevonskogo Meditsinskogo Instituta (Proceedings of the Yerevan Medical Institute), 2, 241–245.

[risa13397-bib-0005] Bartsch, H. , Malaveille, C. , Barbin, A. , & Planche, G. (1979). Mutagenic and alkylating metabolites of halo‐ethylenes, chlorobutadienes and dichlorobutenes produced by rodent or human liver tissues. Evidence for oxirane formation by P450‐linked microsomal mono‐oxygenases. Archives of Toxicology, 41(4), 249–277. 373707 10.1007/BF00296896

[risa13397-bib-0006] Bogen, K. T. , Benson, J. M. , Yost, G. S. , Morris, J. B. , Dahl, A. R. , Clewell, H. J. 3rd , … Omiecinski, C. J. (2008). Naphthalene metabolism in relation to target tissue anatomy, physiology, cytotoxicity and tumorigenic mechanism of action. Regulatory Toxicology and Pharmacology, 51(2), 27–36. 10.1016/j.yrtph.2007.10.018 PMC4030291 18191315

[risa13397-bib-0007] Bond, J. A. , Himmelstein, M. W. , Seaton, M. , Boogaard, P. , & Medinsky, M. A. (1996). Metabolism of butadiene by mice, rats, and humans: A comparison of physiologically based toxicokinetic model predictions and experimental data. Toxicology, 113, 48–54. 8901882 10.1016/0300-483x(96)03426-9

[risa13397-bib-0008] Bukowski, J. A. (2009). Epidemiologic evidence for chloroprene carcinogenicity: Review of study quality and its application to risk assessment. Risk Analysis, 29(9), 1203–1216. 10.1111/j.1539-6924.2009.01254.x 19558388

[risa13397-bib-0009] Bulbulyan, M. A. , Changuina, O. V. , Zaridze, D. G. , Astashevsky, S. V. , Colin, D. , & Boffetta, P. (1998). Cancer mortality among Moscow shoe workers exposed to chloroprene (Russia). Cancer Causes & Control, 9, 381–387. 9794169 10.1023/a:1008863516506

[risa13397-bib-0010] Bulbulyan, M. A. , Margaryan, A. G. , Ilychova, S. A. , Astashevsky, S. V. , Uloyan, S. M. , Cogan, V. Y. , & Zaridze, D. G. (1999). Cancer incidence and mortality in a cohort of chloroprene workers from Armenia. International Journal of Cancer, 81(1), 31–33. 10077148 10.1002/(sici)1097-0215(19990331)81:1<31::aid-ijc6>3.0.co;2-t

[risa13397-bib-0011] Buzard, G. S. (1996). Studies of oncogene activation and tumor suppressor gene inactivation in normal and neoplastic rodent tissue. Mutation Research, 365(1–3), 43–58. 8898988 10.1016/s0165-1110(96)90011-1

[risa13397-bib-0012] Checkoway, H. , Dell, L. D. , Boffetta, P. , Gallagher, A. E. , Crawford, L. , Lees, P. S. , & Mundt, K. A. (2015). Formaldehyde exposure and mortality risks from acute myeloid leukemia and other lymphohematopoietic malignancies in the US national cancer institute cohort study of workers in formaldehyde industries. Journal of Occupational and Environmental Medicine, 57(7), 785–794. 10.1097/JOM.0000000000000466 26147546 PMC4479664

[risa13397-bib-0013] Checkoway, H. , Pearce, N. , & Kriebel, D. (2004). Research methods in occupational epidemiology (2nd ed., Vol. 34). New York, NY: Oxford University Press.

[risa13397-bib-0014] Chen, Z. M. , Liu, B. Q. , Boreham, J. , Wu, Y. P. , Chen, J. S. , & Peto, R. (2003). Smoking and liver cancer in China: Case‐control comparison of 36,000 liver cancer deaths vs. 17,000 cirrhosis deaths. International Journal of Cancer, 107(1), 106–112. 10.1002/ijc.11342 12925964

[risa13397-bib-0015] Chen, H. , Sun, J. , Jiang, H. , Wang, X. , Wu, L. , Wu, W. , & Wang, Q. (2017). Inferring alcoholism SNPs and regulatory chemical compounds based on ensemble Bayesian network. Combinatorial Chemistry & High Throughput Screening, 20(2), 107–115. 28000566 10.2174/1386207319666161220114917

[risa13397-bib-0016] Clary, J. J. (1977). Toxicity of chloroprene 1,3‐dichlorobutene‐2 and 1,4‐dichlorobutene‐2. Environmental Health Perspectives, 21, 269–274. 348457 10.1289/ehp.7721269 PMC1475317

[risa13397-bib-0017] Clewell, H. J. III , Campbell, J. L. , Van Landingham, C. , Franzen, A. , Yoon, M. , Dodd, D. E. , … Gentry, P. R. (2019). Incorporation of *in vitro* metabolism data and physiologically based pharmacokinetic modeling in a risk assessment for chloroprene. Submitted to Inhalation Toxicology. 10.1080/08958378.2020.1715513 31992090

[risa13397-bib-0018] Colonna, M. , & Laydevant, G. (2001). A cohort study of workers exposed to chloroprene in the department of Isere, France. Chemico‐Biological Interactions, 135–136, 505–514. 10.1016/s0009-2797(01)00185-5 11397409

[risa13397-bib-0019] Cottrell, L. , Golding, B. T. , Munter, T. , & Watson, W. P. (2001). In vitro metabolism of chloroprene: Species differences, epoxide stereochemistry and a de‐chlorination pathway. Chemical Research in Toxicology, 14(11), 1552–1562. 11712914 10.1021/tx0155404

[risa13397-bib-0020] Drasgow, F. (1986). Polychoric and polyserial correlations In Kotz S. & Johnson N. L. (Eds.), Encyclopedia of statistical sciences (Vol. 7, pp. 68–74). New York, NY: John Wiley & Sons, Inc.

[risa13397-bib-0021] Drevon, C. , & Kuroki, T. (1979). Mutagenicity of vinyl chloride, vinylidene chloride and chloroprene in V79 Chinese hamster cells. Mutation Research, 67(2), 173–182. 470972 10.1016/0165-1218(79)90129-0

[risa13397-bib-0022] Eckert, E. , Leng, G. , Gries, W. , & Goen, T. (2012). A method for the simultaneous determination of mercapturic acids as biomarkers of exposure to 2‐chloroprene and epidhlorohydrin in human urine. Journal of Chromatography, B. Analytical Technologies in the Biomedical and Life Sciences, 889–890, 69–76. 10.1016/j.jchromb.2012.01.032 22342446

[risa13397-bib-0023] Eckert, E. , Leng, G. , Gries, W. , & Goen, T. (2013). Excretion of mercapturic acids in human urine after occupational exposure to 2‐chloroprene. Archives of Toxicology, 87(6), 1095–1102. 23380765 10.1007/s00204-013-1016-6

[risa13397-bib-0024] Environmental Protection Agency (EPA) . (2005a). Guidelines for carcinogen risk asessment. EPA/630/P‐03/001F. Washington, DC: Risk Assessment Forum, U.S. Environmental Protection Agency.

[risa13397-bib-0025] Environmental Protection Agency (EPA) . (2005b). Supplemental guidance for assessing susceptibility from early‐life exposure to carcinogens. EPA/630/R‐03/003F. Washington, DC: Risk Assessment Forum, U.S. Environmental Protection Agency.

[risa13397-bib-0026] Environmental Protection Agency (EPA) . (2009). Risk Assessment guidance of superfund. Volume I: Human health evaluation manual (Part F, Supplemental Guidance for Inhalation Risk Assessment). EPA‐540‐R‐070‐002, OSWER 9285.7‐82. Washington, DC: Office of Superfund Remediation & Technology Innovation, Environmental Protection Agency.

[risa13397-bib-0027] Environmental Protection Agency (EPA) . (2010). Toxicological review of chloroprene (CAS No. 126‐99‐8) In support of summary information on the Integrated Risk information System (IRIS). (EPA/635/R‐09/010F). Washington, DC: National Center for Environmental Assessment. Office of Research and Development.

[risa13397-bib-0028] Environmental Protection Agency (EPA) . (2011). National air toxics assessment. 2011 NATA: Assessment results. Washington, DC: U.S. Environmental Protection Agency. Retrieved from https://www.epa.gov/national-air-toxics-assessment/2011-nata-assessment-results#pollutant

[risa13397-bib-0029] Garcia, E. , Hurley, S. , Nelson, D. O. , Hertz, A. , & Reynolds, P. (2015). Hazardous air pollutants and breast cancer risk in California teachers: A cohort study. Environmental Health: A Global Access Science Source, 14, 14. 25636809 10.1186/1476-069X-14-14 PMC4417287

[risa13397-bib-0030] Gervasi, P. G. , & Longo, V. (1990). Metabolism and mutagenicity of isoprene. Environmental Health Perspectives, 86, 85–87. 2401275 10.1289/ehp.908685 PMC1567766

[risa13397-bib-0031] Guo, Y. , & Xing, Y. (2016). Weighted gene co‐expression network analysis of pneumocytes under exposure to a carcinogenic dose of chloroprene. Life Sciences, 151, 339–347. 26916823 10.1016/j.lfs.2016.02.074

[risa13397-bib-0032] Himmelstein, M. W. , Carpenter, S. C. , Evans, M. V. , Hinderliter, P. M. , & Kenyon, E. M. (2004). Kinetic modeling of beta‐chloroprene metabolism: II. The application of physiologically based modeling for cancer dose response analysis. Toxicological Sciences, 79(1), 28–37. 10.1093/toxsci/kfh096 14976335

[risa13397-bib-0033] Himmelstein, M. W. , Carpenter, S. C. , & Hinderliter, P. M. (2004). Kinetic modeling of beta‐chloroprene metabolism: I. In vitro rates in liver and lung tissue fractions from mice, rats, hamsters, and humans. Toxicological Sciences, 79(1), 18–27. 10.1093/toxsci/kfh092 14976339

[risa13397-bib-0034] Himmelstein, M. W. , Carpenter, S. C. , Hinderliter, P. M. , Snow, T. A. , & Valentine, R. (2001). The metabolism of beta‐chloroprene: Preliminary in‐vitro studies using liver microsomes. Chemico‐Biological Interactions, 135–136, 267–284. 10.1016/s0009-2797(01)00214-9 11397396

[risa13397-bib-0035] Himmelstein, M. W. , Gladnick, N. L. , Donner, E. M. , Snyder, R. D. , & Valentine, R. (2001). In vitro genotoxicity testing of (1‐chloroethenyl)oxirane, a metabolite of beta‐chloroprene. Chemico‐Biological Interactions, 135–136, 703–713. 10.1016/s0009-2797(01)00203-4 11397425

[risa13397-bib-0036] International Institute of Synthetic Rubber Producers (IISRP) . (2009). Chloroprene: Blood Concentration Toxicokinetics in Female Mice by Single and Repeated Inhalation Exposure, IISRP‐12828–1388.

[risa13397-bib-0037] Lee, Y. C. , Cohet, C. , Yang, Y. C. , Stayner, L. , Hashibe, M. , & Straif, K. (2009). Meta‐analysis of epidemiologic studies on cigarette smoking and liver cancer. International Journal of Epidemiology, 38(6), 1497–1511. 10.1093/ije/dyp280 19720726

[risa13397-bib-0038] Leet, T. L. , & Selevan, S. (1982). Mortality analysis of workers exposed to chloroprene. Cincinnati, OH: National Institute for Occupational Safety and Health; Center for Disease Control, Department of Health and Human Services.

[risa13397-bib-0039] Levi, F. , Lucchini, F. , Negri, E. , Boyle, P. , & La Vecchia, C. (2004). Cancer mortality in Europe, 1995–1999, and an overview of trends since 1960. International Journal of Cancer, 110, 155–169. 15069676 10.1002/ijc.20097

[risa13397-bib-0040] Lewis, D. F. , Ioannides, C. , & Parke, D. V. (1996). COMPACT and molecular structure in toxicity assessment: A prospective evaluation of 30 chemicals currently being tested for rodent carcinogenicity by the NCI. Environmental Health Perspectives, 104(Suppl 5), 1011–1016. 8933049 10.1289/ehp.96104s51011 PMC1469712

[risa13397-bib-0041] Li, S. Q. , Dong, Q. N. , Liu, Y. Q. , & Liu, Y. G. (1989). Epidemiologic study of cancer mortality among chloroprene workers. Biomedical and Environmental Sciences, 2(2), 141–149. 2590499

[risa13397-bib-0042] Lloyd, J. W. (1976). Cancer risks among workers exposed to chloroprene. Annals of the New York Academy of Sciences, 271, 91–93. 1069543 10.1111/j.1749-6632.1976.tb23097.x

[risa13397-bib-0043] London, W. T. , & McGlynn, K. A. (2006). Liver cancer In Schottenfeld D. & Fraumeni J. F. Jr. (Eds.), Cancer epidemiology and prevention (3rd ed., pp. 763–786). New York, NY: Oxford University Press, Inc.

[risa13397-bib-0044] Maniscalco L., French J., Rosales C., Lefante C., Hsieh M., Zhang L., … Wu X. C. (Eds.) (2018). Cancer in Louisiana (Vol. 33, pp. 2011–2015). New Orleans, LA: Louisiana Tumor Registry.

[risa13397-bib-0045] Maniscalco L., Zhang L., Yi Y., Lefante C., Rosales C., Hsieh M. C., & Wu X. C. (Eds.) (2019). Cancer incidence in Louisiana by census tract (pp. 2005–2015). New Orleans, LA: Louisiana Tumor Registry Retrieved from https://sph.lsuhsc.edu/louisiana-tumor-registry/data-usestatistics/monographs-publications/cancer-incidence-louisiana-census-tract/

[risa13397-bib-0046] Marsh, G. M. , Youk, A. O. , Buchanich, J. M. , Cunningham, M. , Esmen, N. A. , Hall, T. A. , & Phillips, M. L. (2007a). Mortality patterns among industrial workers exposed to chloroprene and other substances. I. General mortality patterns. Chemico‐Biological Interactions, 166(1–3), 285–300. 10.1016/j.cbi.2006.08.011 16999943

[risa13397-bib-0047] Marsh, G. M. , Youk, A. O. , Buchanich, J. M. , Cunningham, M. , Esmen, N. A. , Hall, T. A. , & Phillips, M. L. (2007b). Mortality patterns among industrial workers exposed to chloroprene and other substances. II. Mortality in relation to exposure. [Research Support, Non‐U.S. Gov't]. Chemico‐Biological Interactions, 166(1–3), 301–316. 10.1016/j.cbi.2006.08.012 17007827

[risa13397-bib-0048] Melnick, R. L. , Elwell, M. R. , Roycroft, J. H. , Chou, B. J. , Ragan, H. A. , & Miller, R. A. (1996). Toxicity of inhaled chloroprene (2‐chloro‐1,3‐butadiene) in F344 rats and B6C3F(1) mice. Toxicology, 108(1–2), 79–91. 8644121 10.1016/0300-483x(95)03286-o

[risa13397-bib-0049] Melnick, R. L. , & Kohn, M. C. (2000). Dose‐response analyses of experimental cancer data. Drug Metabolism Reviews, 32(2), 193–209. 10774775 10.1081/dmr-100100572

[risa13397-bib-0050] Morgan, D. L. (1997). Short‐term inhalation carcinogenesis study of chloroprene in transgenic animal. Research Triangle, NC: National Institute of Environmental Health Sciences. Retrieved from https://www.niehs.nih.gov/

[risa13397-bib-0051] National Research Council (NRC) . (2011). Review of the environmental protection agency's draft IRIS assessment of formaldehyde. Washington, DC: The National Academies Press. 25032397

[risa13397-bib-0052] National Research Council (NRC) . (2014). Review of EPA's integrated risk information system (IRIS) process. Washington, DC: The National Academies Press. 25101400

[risa13397-bib-0053] National Toxicology Program (NTP) . (1998). Toxicology and carcinogenesis studies of chloroprene (CAS No. 126‐99‐8) in F344 rats and B6C3F1 mice (inhalation studies). Report No. NTP TR 467 (pp. 1–372). Research Triangle Park, NC: National Toxicology Program. 12579206

[risa13397-bib-0054] National Toxicology Program (NTP) . (2016). Fourteenth Edition of Report on Carcinogens. Chloroprene (CAS No. 126‐99‐8). Retrieved from https://ntp.niehs.nih.gov/pubhealth/roc/index-1.html#toc1

[risa13397-bib-0055] Olsson, U. (1979). Maximum likelihood estimation of the polychoric correlation coefficient. Psychometrika, 44(4), 443–460.

[risa13397-bib-0056] Olsson, U. , Drasgow, F. , & Dorans, N. (1982). The polyserial correlation coefficient. Psychometrika, 47(3), 337–347.

[risa13397-bib-0057] Pagan, I. (2007). Chloroprene: Overview of studies under consideration for the development of an IRIS assessment. Chemico‐Biological Interactions, 166(1–3), 341–351. 10.1016/j.cbi.2006.12.001 17234169

[risa13397-bib-0058] Pell, S. (1978). Mortality of workers exposed to chloroprene. Journal of Occupational Medicine, 20(1), 21–29. 621591 10.1097/00043764-197801000-00006

[risa13397-bib-0059] Rannug, A. H. , & Ostman, C. (1983). Application of mutagenicity tests for detection and source assessment of genotoxic agents in the rubber work atmosphere. Short‐term bioassays in the analysis of complex environmental mixtures III. Environmental Science Research, 27, 541–553.

[risa13397-bib-0060] Rice, J. M. , & Boffetta, P. (2001). 1,3‐Butadiene, isoprene and chloroprene: Reviews by the IARC monographs programme, outstanding issues, and research priorities in epidemiology. Chemico‐Biological Interactions, 135–136, 11–26. 10.1016/s0009-2797(01)00175-2 11397378

[risa13397-bib-0061] Rickert, A. , Hartung, B. , Kardel, B. , Teloh, J. , & Daldrup, T. (2012). A fatal intoxication by chloroprene. Forensic Science International, 215, 1–3. 21511420 10.1016/j.forsciint.2011.03.029

[risa13397-bib-0062] Sanotskii, I. V. (1976). Aspects of the toxicology of chloroprene: Immediate and long‐term effects. [Review]. Environmental Health Perspectives, 17, 85–93. 799963 10.1289/ehp.761785 PMC1475249

[risa13397-bib-0063] SAS Institute Inc . (2016). Base SAS 9.4 Procedures guide: Statistical procedures, Fifth Edition. Cary, NC: SAS Institute Inc. Retrieved from https://support.sas.com/documentation/cdl/en/procstat/70116/PDF/default/procstat.pdf

[risa13397-bib-0064] Shelby, M. D. (1990). Results of NTP‐sponsored mouse cytogenetic studies on 1,3‐butadiene, isoprene, and chloroprene. Environmental Health Perspectives, 86, 71–73. 2401274 10.1289/ehp.908671 PMC1567722

[risa13397-bib-0065] Shelby, M. D. , & Witt, K. L. (1995). Comparison of results from mouse bone marrow chromosome aberration and micronucleus tests. [Comparative Study Research Support, U.S. Gov't, P.H.S.]. Environmental and Molecular Mutagenesis, 25(4), 302–313. 7607185 10.1002/em.2850250407

[risa13397-bib-0066] Sills, R. C. , Hong, H. L. , Melnick, R. L. , Boorman, G. A. , & Devereux, T. R. (1999). High frequency of codon 61 K‐ras A–>T transversions in lung and Harderian gland neoplasms of B6C3F1 mice exposed to chloroprene (2‐chloro‐1,3‐butadiene) for 2 years, and comparisons with the structurally related chemicals isoprene and 1,3‐butadiene [Comparative Study]. Carcinogenesis, 20(4), 657–662. 10223196 10.1093/carcin/20.4.657

[risa13397-bib-0067] Stuver, S. , & Trichopoulos, D. (2008). Cancer of the liver and biliary tract, Chapter 12. In Adami H. O., Hunter D., & Trichopoulos D. (Eds.), Textbook of cancer epidemiology (2nd ed., pp. 308–332). New York, NY: Oxford University Press.

[risa13397-bib-0068] Sutherland, K. M. , Edwards, P. C. , Combs, T. J. , & Van Winkle, L. S. (2012). Sex differences in the development of airway epithelial tolerance to naphthalene. American Journal of Physiology‐Lung Cellular & Molecular Physiology, 302(1), L68–L81. 22003090 10.1152/ajplung.00089.2011 PMC3349371

[risa13397-bib-0069] Thomas, R. S. , Himmelstein, M. W. , Clewell, H. J. , Yang, Y. , Healy, E. , Black, M. B. , & Andersen, M. E. (2013). Cross‐species transcriptomic analysis of mouse and rat lung exposed to chloroprene. Toxicological Sciences, 131(2), 629–640. 23125180 10.1093/toxsci/kfs314

[risa13397-bib-0070] Tice, R. R. (1988). The cytogenetic evaluation of in vivo genotoxic and cytotoxic activity using rodent somatic cells. [Research Support, U.S. Gov't, Non‐P.H.S. Research Support, U.S. Gov't, P.H.S.]. Cell Biology and Toxicology, 4(4), 475–486. 3228715 10.1007/BF00117775

[risa13397-bib-0071] Tice, R. R. , Boucher, R. , Luke, C. A. , Paquette, D. E. , Melnick, R. L. , & Shelby, M. D. (1988). Chloroprene and isoprene: Cytogenetic studies in mice. Mutagenesis, 3(2), 141–146. 3288837 10.1093/mutage/3.2.141

[risa13397-bib-0072] Trochimowicz, H. J. , Loser, E. , Feron, V. J. , Clary, J. J. , & Valentine, R. (1998). Chronic inhalation toxicity and carcinogenicity studies on beta‐chloroprene in rats and hamsters. Inhalation Toxicology, 10(5), 443–472.

[risa13397-bib-0073] Valentine, R. , & Himmelstein, M. W. (2001). Overview of the acute, subchronic, reproductive, developmental and genetic toxicology of beta‐chloroprene. Chemico‐Biological Interactions, 135–136, 81–100. 10.1016/s0009-2797(01)00218-6 11397383

[risa13397-bib-0074] Van Winkle, L. S. , Gunderson, A. D. , Shimizu, J. A. , Baker, G. L. , & Brown, C. D. (2002). Gender differences in naphthalene metabolism and naphthalene‐induced acute lung injury. American Journal of Physiology, 282(5), L1122–L1134. 11943679 10.1152/ajplung.00309.2001

[risa13397-bib-0075] Wadugu, B. A. , Ng, C. , Bartley, B. L. , Rowe, R. J. , & Millard, J. T. (2010). DNA interstrand cross‐linking activity of (1‐Chloroethenyl)oxirane, a metabolite of beta‐chloroprene. Chemical Research in Toxicology, 23(1), 235–239. 20030381 10.1021/tx9003769 PMC2814250

[risa13397-bib-0076] Westphal, G. A. , Blaszkewicz, M. , Leutbecher, M. , Muller, A. , Hallier, E. , & Bolt, H. M. (1994). Bacterial mutagenicity of 2‐chloro‐1,3‐butadiene (chloroprene) caused by decomposition products. Archives of Toxicology, 68(2), 79–84. 8179486 10.1007/s002040050038

[risa13397-bib-0077] Willems, M. (1980). Evaluation of β‐chloroprene and four chloroprene dimmers in the Ames test by atmospheric exposure of the tester strains. Final Report No. R‐6392 by Central Institute for Nutrition and Food research for the Joint Industry Committee on Chloroprene.

[risa13397-bib-0078] Yamada, T. , Kondo, M. , Miyata, K. , Ogata, K. , Kushida, M. , Sumida, K. , … Cohen, S. M. (2017). An evaluation of the human relevance of the tumors observed in female mice treated with permethrin based on mode of action. Toxicological Sciences, 157(2), 465–486. 28431163 10.1093/toxsci/kfx066

[risa13397-bib-0079] Yang, Y. , Himmelstein, M. W. , & Clewell, H. J. (2012). Kinetic modeling of beta‐chloroprene metabolism: Probabilistic in vitro‐in vivo extrapolation of metabolism in the lung, liver and kidneys of mice, rats and humans. Toxicology in Vitro, 26(6), 1047–1055. 10.1016/j.tiv.2012.04.004 22543297

[risa13397-bib-0080] Zaridze, D. , Bulbulyan, M. , Changuina, O. , Margaryan, A. , & Boffetta, P. (2001). Cohort studies of chloroprene‐exposed workers in Russia. Chemico‐Biological Interactions, 135, 487–503. 11397408 10.1016/s0009-2797(01)00184-3

[risa13397-bib-0081] Zeiger, E. , Anderson, B. , Haworth, S. , Lawlor, T. , Mortelmans, K. , & Speck, W. (1987). Salmonella mutagenicity tests: III. Results from the testing of 255 chemicals. Environmental Mutagenesis, 9(Suppl 9), 1–109. 3552650

[risa13397-bib-0082] Zhang, Y. P. , Sussman, N. , Macina, O. T. , Rosenkranz, H. S. , & Klopman, G. (1996). Prediction of the carcinogenicity of a second group of organic chemicals undergoing carcinogenicity testing. Environmental Health Perspectives, 104(Suppl 5), 1045–1050. 8933053 10.1289/ehp.96104s51045 PMC1469705

